# Modeling the emergence of migratory corridors and foraging hot spots of the green sea turtle

**DOI:** 10.1002/ece3.5552

**Published:** 2019-08-18

**Authors:** Mayeul Dalleau, Stephanie Kramer‐Schadt, Yassine Gangat, Jérôme Bourjea, Gilles Lajoie, Volker Grimm

**Affiliations:** ^1^ Centre d'Etude et de Découverte des Tortues Marines (CEDTM) Saint Leu/La Réunion France; ^2^ Department of Ecological Dynamics Leibniz Institute for Zoo and Wildlife Research Berlin Germany; ^3^ Department of Ecology Technische Universität Berlin Berlin Germany; ^4^ LIM‐IREMIA, EA2525 University of La Réunion, PTU Sainte‐Clotilde/La Réunion France; ^5^ Institut Français de Recherche pour l'Exploitation de la Mer MARBEC Université de Montpellier CNRS Ifremer IRD Sète Cedex France; ^6^ UMR Espace‐Dev University of La Réunion Saint‐Denis France; ^7^ Department of Ecological Modelling Helmholtz Centre for Environmental Research – UFZ Leipzig Germany; ^8^ Department of Plant Ecology and Nature Conservation University of Potsdam Potsdam‐Golm Germany; ^9^ German Centre for Integrative Biodiversity Research (iDiv) Halle‐Jena‐Leipzig Leipzig Germany

**Keywords:** connectivity, corridors, individual‐based model, migration, movement, sea turtle

## Abstract

Environmental factors shape the spatial distribution and dynamics of populations. Understanding how these factors interact with movement behavior is critical for efficient conservation, in particular for migratory species. Adult female green sea turtles, *Chelonia mydas*, migrate between foraging and nesting sites that are generally separated by thousands of kilometers. As an emblematic endangered species, green turtles have been intensively studied, with a focus on nesting, migration, and foraging. Nevertheless, few attempts integrated these behaviors and their trade‐offs by considering the spatial configurations of foraging and nesting grounds as well as environmental heterogeneity like oceanic currents and food distribution. We developed an individual‐based model to investigate the impact of local environmental conditions on emerging migratory corridors and reproductive output and to thereby identify conservation priority sites. The model integrates movement, nesting, and foraging behavior. Despite being largely conceptual, the model captured realistic movement patterns which confirm field studies. The spatial distribution of migratory corridors and foraging hot spots was mostly constrained by features of the regional landscape, such as nesting site locations, distribution of feeding patches, and oceanic currents. These constraints also explained the mixing patterns in regional forager communities. By implementing alternative decision strategies of the turtles, we found that foraging site fidelity and nesting investment, two characteristics of green turtles' biology, are favorable strategies under unpredictable environmental conditions affecting their habitats. Based on our results, we propose specific guidelines for the regional conservation of green turtles as well as future research suggestions advancing spatial ecology of sea turtles. Being implemented in an easy to learn open‐source software, our model can coevolve with the collection and analysis of new data on energy budget and movement into a generic tool for sea turtle research and conservation. Our modeling approach could also be useful for supporting the conservation of other migratory marine animals.

## Graphical abstract

Our model represents the migration of green sea turtles in the southwest Indian Ocean between nesting and foraging sites. It can cover thousands of km and is contratined by the regional landscape and oceanic currents. Emerging migration corridors were identified, which can be used for identifying priority sites for conservation.

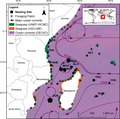

## 1 INTRODUCTION

Many species migrate to exploit resources heterogeneously distributed in space and time (Jorgensen, Dunlop, Opdal, & Fiksen, 2008). Individuals must allocate these resources internally to growth, survival, and reproduction in a way that maximizes their fitness (Martin, Jager, Preuss, Nisbet, & Grimm, 2013; Roff, 2002; Sibly et al., 2013; Varpe, Jørgensen, Tarling, & Fiksen, 2008). Animal migration costs must therefore be balanced by fitness benefits (Milner‐Gulland, Fryxell, & Sinclair, 2011). Consequently, even small changes, for example, in the quality of breeding or feeding patches can significantly influence long‐term population survival (Fiksen & Jorgensen, 2011; Taylor & Norris, 2010).

**Figure 1:**
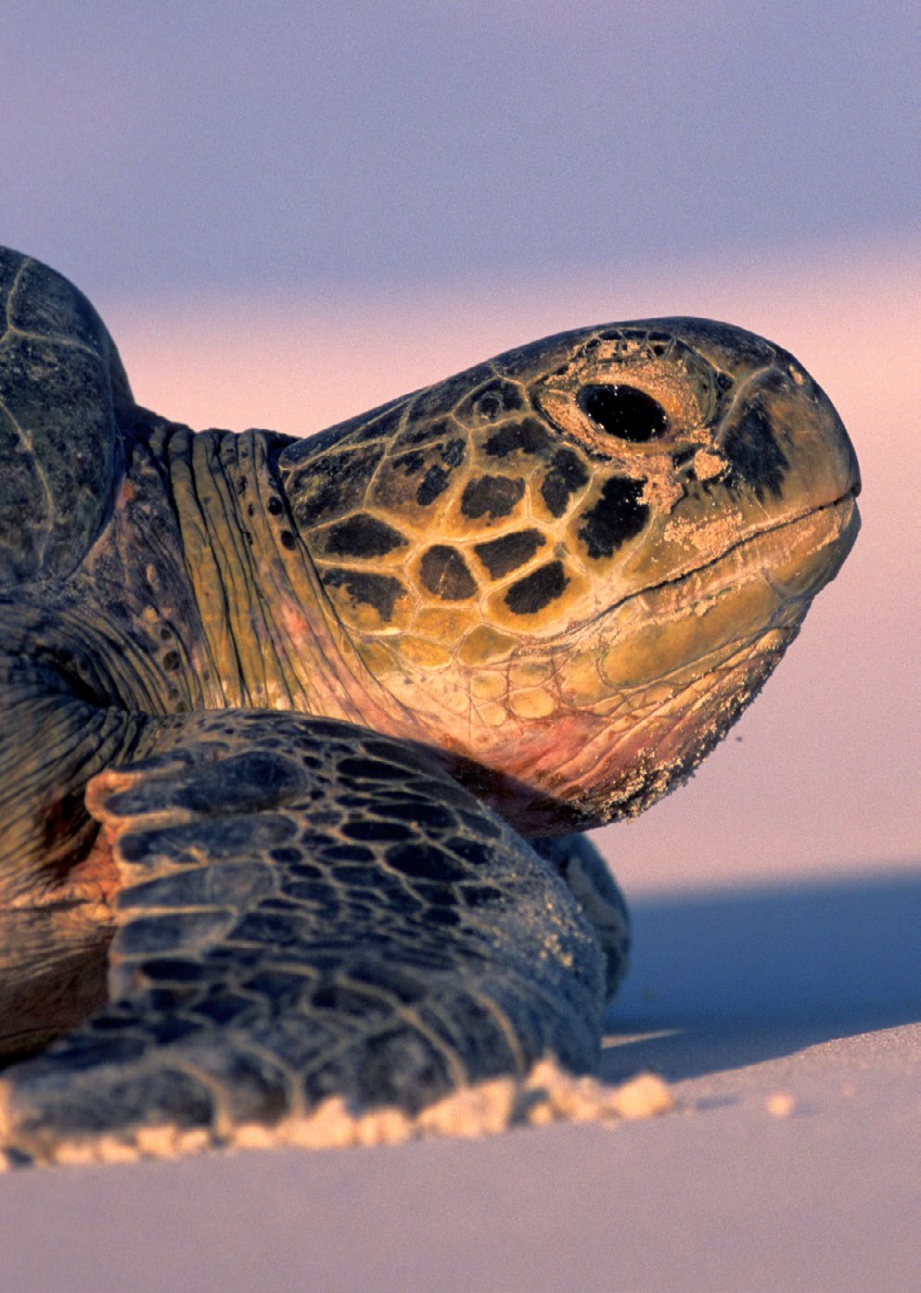
The green sea turtle, *Chelonia mydas* (photo: J. Bourjea/Ifremer)

**Figure 2:**
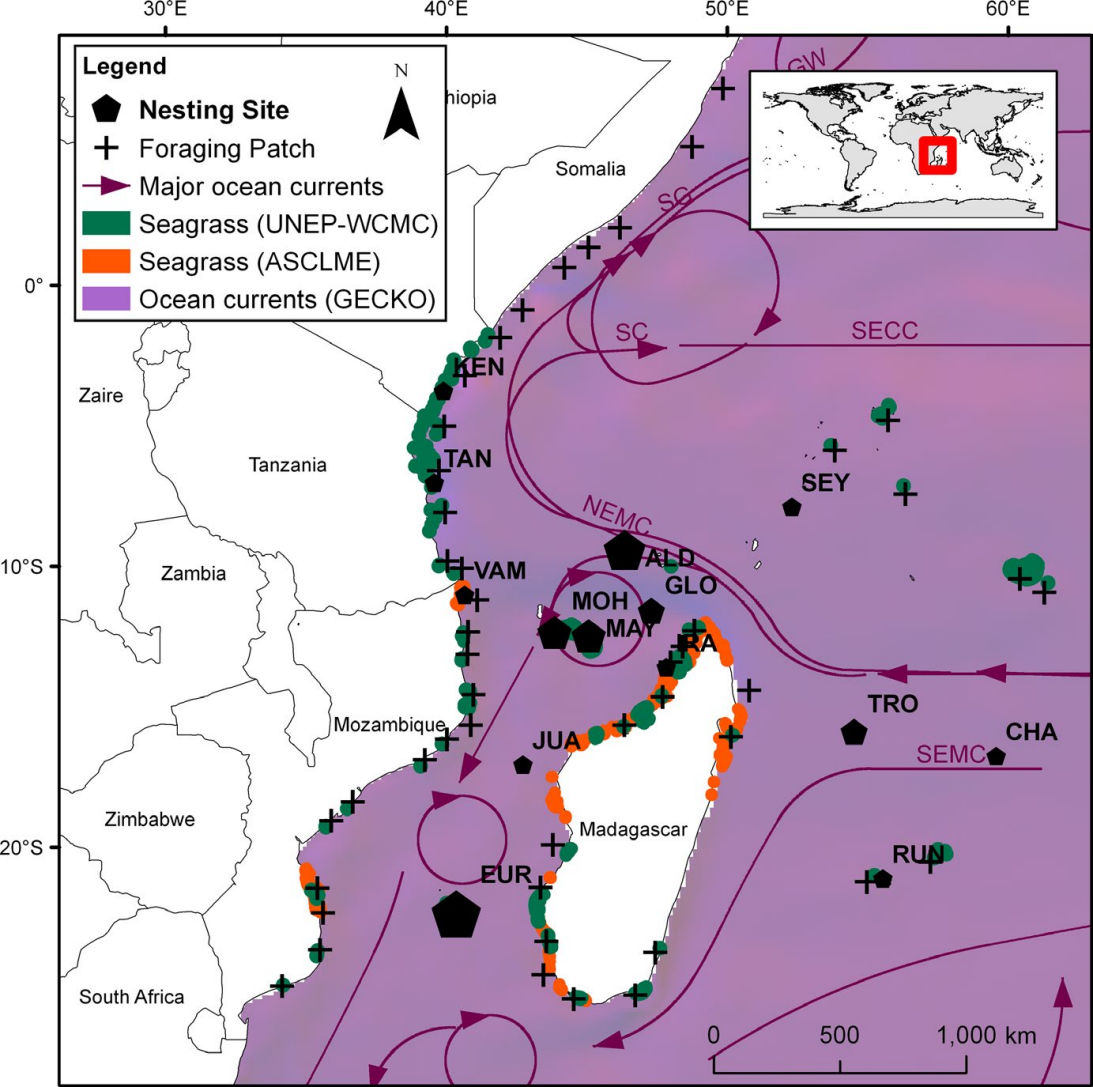
Overview of the SWIO landscape. Black pentagons represent nesting sites: ALD, Aldabra; EUR, Europa; IRA, Iranja; MAY, Mayotte; MOH, Mohéli; TRO, Tromelin; VAM, Vamizi. Size of nesting site is proportional to nesting number of females. Black crosses represent locations of feeding patches. Arrows indicate major oceanic currents (Schott, Xie, & McCreary, 2009): GW, Great Whirl. Red and blue levels indicate mean annual oceanic current intensities; NEMC, North Equatorial Madagascar current; SC, Somalia Current; SECC, South Equatorial Counter Current; SEMC, South Equatorial Madagascar Current; SG, Southern Gyre. In the legend of the figure, the acronyms describe the input data sources: United Nations Environment Programme—World Conservation Monitoring Centre (UNEP‐WCMC, Green & Short, 2003), Agulhas and Somali Current Large Marine Ecosystems Project (ASCLME, http://www.asclme.org), Geostrophic and Ekman Current Observatory (GECKO, Sudre et al., 2013)

The green sea turtle, *Chelonia mydas*, is a wide‐ranging species distributed worldwide (Plotkin, 2003; Figure 1) and classified as endangered in the IUCN red list (Seminoff, 2004). As adults, green turtles perform long‐distance migration between feeding and nesting sites, which are generally separated by thousands of kilometers (Godley et al., 2008). They exhibit strong natal philopatry (or natal homing) and tend to nest on the same site that they hatched (Jensen et al., 2019; Lohmann, Witherington, Lohmann, & Salmon, 1997). The southwest Indian Ocean (SWIO) shelters some of the world's major green turtle rookeries (Bourjea, Dalleau, et al., 2015a; Bourjea, Frappier, et al., 2007a; Dalleau et al., 2012; Derville et al., 2015; Lauret‐Stepler et al., 2007; Mortimer, Brandis, Liljevik, Chapman, & Collie, 2011b) that are distributed across the entire region on oceanic islands spread along the Mozambique Channel and the Mascarene plateau (Figure 2). Other minor nesting sites are located on continental islands and shores on the coast of Madagascar and East Africa (Bourjea, Ciccione, & Ratsimbazafy, 2006; Garnier et al., 2012). Seagrass beds, the main component of adult green turtle diet (Bjorndal, 1997), extend almost continuously over the east African coast from Mozambique to Somalia and over the western coast of Madagascar (Figure 2; Gullström et al., 2002), and foraging green turtles are observed in all countries of the SWIO hosting seagrass beds (Ballorain et al., 2010; Fulanda et al., 2007; Muir, 2005; Okemwa, Nzuki, & Mueni, 2004; Williams, Pierce, Rohner, Fuentes, & Hamann, 2017). A tracking study (Dalleau, 2013) demonstrated that (a) the northern part of the Mozambique Channel is a major oceanic migration corridor for postnesting green turtles capable to migrate thousands of kilometers, (b) coastal grounds of East Africa and West Madagascar are important foraging sites and migration corridors, (c) turtles from the SWIO nesting sites make extensive use of available foraging habitats of the whole region, and (d) foraging grounds are used by turtles originating from different rookeries of the SWIO.

Threats are highly variable in the SWIO region, which is bordered by countries and provinces of heterogeneous economic levels. The region has been identified as a specific “Regional Management Unit” for the green turtle, that is, a spatially explicit population segments defined by biogeographical data of this species (Wallace, DiMatteo, et al., 2010a). Long‐term local protection at nesting sites is an important component of sea turtle conservation (Chaloupka et al., 2008). Nevertheless, adult green turtles spend most of their lifetime on foraging grounds where they are exposed to important threats such as direct exploitation of eggs, meat, and shells or fisheries interaction (Wallace, Lewison, et al., 2010b), especially in the SWIO (Bourjea, 2015; Temple et al., 2018; Williams, Pierce, Fuentes, & Hamann, 2016) where for instance more than between 10,000 and 16,000 green turtles were estimated to be captured by the local artisanal fishery to be sold in local markets for consumption each year only in a portion of the south west coast of Madagascar (Humber, Godley, Ramahery, & Broderick, 2011). Thus, conservation plans can only be efficient with coordinated protection measures encompassing the whole spatial scale of sea turtle's distribution. To focus conservation efforts where they are most required and efficient, it is an urgent need to understand the factors that govern the spatial dynamics of the species and the life‐history strategies that lead to effective cycles of foraging, migration, and nesting.

Several concepts exist to describe how resource patches are most efficiently exploited by animals (Eliassen, Jorgensen, Mangel, & Giske, 2009). An example is the Marginal Value Theorem (Charnov, 1976) that predicts that a forager should leave a patch when its food intake drops below the average food intake on all other patches. As another example, the theory of Ideal Free Distribution (Fretwell & Lucas, 1970) predicts that the proportion of individuals exploiting different given resource patches should be proportional to the patches' resource levels. Nevertheless, these two complementary concepts (and others) make the somewhat unrealistic assumption that foragers can perfectly assess resource levels and heterogeneity over an entire region and respond accordingly (Eliassen et al., 2009; Railsback & Harvey, 2013). Reality often differs from this assumption. For any species, migration toward a feeding patch requires energetically costly movements that might have limited benefits if the target feeding patch is already depleted. It seems reasonable to assume that in the case of sea turtles, individuals have little if any information about the location of the feeding patches that are ideal at a given time. It thus remains an open question to what degree the distribution of turtles on feeding patches is determined by the sites' accessibility, the turtle's knowledge of their location (foraging site fidelity), and distance to the nesting site.

In addition to feeding patch selection, heterogeneous landscapes are also likely to have strong effects on animal's movement patterns and hence the resulting connectivity among feeding and nesting sites (Graf, Kramer‐Schadt, Fernandez, & Grimm, 2007; Olden, Schooley, Monroe, & Poff, 2004; Pe'er & Kramer‐Schadt, 2008; Revilla, Wiegand, Palomares, Ferreras, & Delibes, 2004). Oceanic currents often play a major role in foraging ecology of marine animals (Bost et al., 2009; Chapman et al., 2011), especially oceanographic fronts (Scales et al., 2014), and sea turtles' oceanic movements are directly affected by oceanic currents (Girard, Sudre, Benhamou, Roos, & Luschi, 2006; Luschi, Hays, & Papi, 2003). The early life stage of marine turtles (that can last decades) is oceanic, and the spatial fate is also strongly impacted by oceanic currents and may have consequences that prevail and shape the spatial dynamics of adult stages (Gaspar & Lalire, 2017). Furthermore, terrestrial areas, with the exception of nesting grounds, constitute barriers to sea turtle's migration as well as potential navigational cues (Hays, Broderick, Godley, et al., 2002b). Migratory constraints might then differ drastically for islands surrounded by coastal areas like Taiwan in the China Sea (Cheng, 2000) in comparison with oceanic isolated islands like Ascension Island in the Southern Atlantic Ocean (Luschi et al., 2003).

In summary, successful sea turtle conservation seems to be intrinsically linked to the foraging and migration processes, with natal homing for nesting being one of the key factors driving sea turtle life history. We therefore developed a spatially explicit individual‐based model (Grimm & Railsback, 2005; Railsback & Grimm, 2019) to qualitatively study the spatial dynamics of adult green turtle in the SWIO.

Individual‐based modeling, in a large sense, has been used before to address various aspects of sea turtle ecology. A first kind of IBM, that was commonly implemented, concerns the spatial fate of hatchlings during their first years in the open ocean. Lagrangian modeling of passive drift trajectories has allowed predicting the spatial cycle of juvenile sea turtles (Blumenthal et al., 2009; Godley et al., 2010; Hays, Fossette, Katselidis, Mariani, & Schofield, 2010; Putman & Naro‐Maciel, 2013). Limits of passive drift are, however, of concern, and models including active swimming behavior were developed. Still, movement rules remained fairly simple and consisted either of random movement (Gaspar et al., 2012; Putman, Scott, Verley, Marsh, & Hays, 2012a) or movement oriented along a gradient of environmental variables or magnetic fields (Putman, Verley, Shay, & Lohmann, 2012b). More recently dispersal affected by oceanic currents and habitat features was modeled for the western Pacific leatherback turtle (Gaspar & Lalire, 2017), as well as the effect of multiple cues on the homing behavior of individual green sea turtles (Painter & Plochocka, 2019). These kinds of IBMs remained focused on movement and did not consider demographic processes such as survival or reproductive output.

Contrastingly, IBMs were also used to represent population dynamics of sea turtles (Mazaris, Broder, & Matsinos, 2006; Mazaris, Fiksen, & Matsinos, 2005; Mazaris & Matsinos, 2006; Piacenza, Richards, & Heppell, 2017), but in these cases, movement was not explicitly implemented. Another type of IBMs was used to study nesting population dynamics such as consequences of variable remigration intervals on sea turtles' nesting numbers (Hays, 2000; Neeman, Spotila, & O'Connor, 2015) or how changes in biological processes can influence population recovery and assessments (Piacenza et al., 2017). Also in these models, movement was also not explicitly implemented.

Our model explicitly represents movement of thousands of sea turtles, but we do not include demographic processes and hence population dynamics. The main purpose of our model is to better understand how the features of the regional landscape, such as nesting site locations, distribution of feeding patches, and oceanic currents, constrain the migratory and foraging patterns of green turtles and to devise implications for the conservation of the species in the region. We implemented alternative foraging and nesting strategies across the entire parameter range, expressing qualitative strategies from being risk prone to risk averse. The model then allowed assessing the influence and sensitivity of different foraging and nesting strategies in concert with feeding patch disturbance on the reproductive output of rookeries.

## 2 METHODS

### 2.1 Life cycle of green turtles

The green turtle's life begins in the sand of the natal beach. After emerging from the nests, sea turtles' hatchlings join oceanic waters and drift with the currents (Carr, 1986). They remain in oceanic waters for years in a stage known as oceanic juvenile stage before recruiting in neritic habitats (Musick & Limpus, 1997). Conditions of recruitment and criteria of site selection remain poorly understood but recruitment zones are often fairly distant from the natal beach (Naro‐Maciel, Becker, Lima, Marcovaldi, & DeSalle, 2007). At this stage, known as the neritic juvenile stage, green turtle's trophic status permanently changes from omnivory to herbivory (Musick & Limpus, 1997). Its main diet thenceforth consists most generally of sea grasses and possibly also of algae (Bjorndal, 1980).

At sexual maturity, sea turtles exhibit strong philopatry, that is, a tendency to breed in the place they were born (Brothers & Putman, 2013; Miller, 1997). Adults consequently migrate back and forth to the natal nesting sites every few years (generally 2–4 years, Troeng & Chaloupka, 2007). The duration between two reproductive cycle, known as the “remigration interval,” varies within and among populations (Heithaus, 2013) and may depend on population recovering status, availability of quality food, or distance to foraging ground (Troeng & Chaloupka, 2007). Green turtles are capital breeders, since they do not feed during reproduction and the reproductive cycle is based on stored energetic reserves. At nesting site, females repeatedly enter the beach shore where they lay eggs in the sand. Postnesting females then migrate to resident neritic foraging areas.

For different sea turtles' species, a site fidelity to foraging areas over multiple reproductive cycles has been observed (Limpus et al., 1992; Marcovaldi et al., 2010; Schofield et al., 2010; Shaver & Rubio, 2008; Tucker, MacDonald, & Seminoff, 2014). In the Mediterranean sea, female green turtles have been tracked migrating to identical foraging locations after successive nesting events (Broderick, Coyne, Fuller, Glen, & Godley, 2007). In the Pacific Ocean, a tagging study also demonstrated foraging site fidelity of female green turtles at different spatial and temporal scale (Read et al., 2014). Nevertheless, change in foraging site has also been observed suggesting that foraging site selection is a plastic behavior (Hays, Hobson, Metcalfe, Righton, & Sims, 2006; Marcovaldi et al., 2010; Shaver & Rubio, 2008).

Green turtles postnesting migrations consist of oceanic and/or coastal movement to preferred foraging areas with relatively direct routes (Godley et al., 2008). Coastal sections along the way may afford foraging opportunities (Cheng, 2000; Godley et al., 2002) but coastlines may also be used to facilitate navigation (Hays, Broderick, Godley, et al., 2002b). Oceanic currents constrain homing and postnesting movements by moving individuals away from their course and lowering the ability to orientate (Cerritelli et al., 2018; Cheng & Wang, 2009; Girard et al., 2006).

### 2.2 Model description

We describe the model following the ODD (Overview, Design concepts, and Details) protocol for individual‐based models (Grimm et al., 2006, 2010). The model was implemented in NetLogo 4.1.3 and released under NetLogo 5.3.1 (Railsback & Grimm, 2019; Wilensky, 1999). The NetLogo program and all data files required to run the model are available under https://www.comses.net/codebases/69863caa-2f8e-4412-a564-a2826d9d38d3/releases/1.0.0/.

#### 2.2.1 Purpose

The proximate purpose of the model is to understand how the features of the SWIO regional landscape, such as nesting site locations, distribution of feeding patches, and oceanic currents, constrain the migratory and foraging patterns of green turtles; its ultimate purpose is to reveal foraging and nesting sites of high conservation value. The model implements the processes of foraging, migration, and nesting to study how they affect the reproductive potential of the main regional rookeries. To go further, the model also explores how different foraging and nesting strategies may affect reproductive output and hence population survival in a heterogeneous landscape.

#### 2.2.2 Entities, state variables, and scales

**Figure 3:**
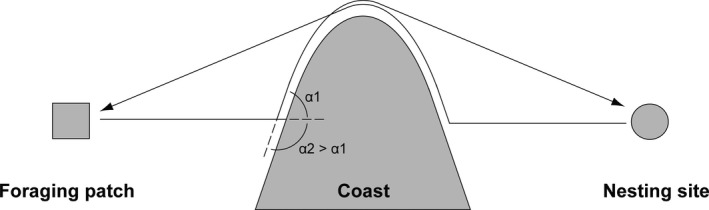
Schematic representation of coast avoidance trajectories. Direction of coast avoidance is determined during first prenesting migration (alternatively foraging migration) by prioritizing the least turning angle (to the left, α1, which is smaller than to the right, α2, in this example). During postnesting migration individual will avoid the coast by turning in the opposite direction compared to prenesting migration (to the right in this example). An individual stops following the coast when it is able to move without obstacle in the direction of the target. This may possibly lead to different trajectories during prenesting and postnesting migration

The entities of the model are adult female green turtles, square grid cells forming a grid that covers the SWIO region (25°E–65°E; 30°S–10°N; Figure 2), and perturbations. The turtles' state variables are location (grid cell), current preferred feeding patch, nesting site, internal state (“feeding”; “prenesting,”, i.e., on the way to the nesting site; “postnesting,” i.e., on the way from the nesting to a feeding patch; “nesting”; “foraging‐migration,” i.e., moving between feeding patches), energy level, foraging strategy, and nesting strategy. Each individual also has a coast avoidance direction that determines whether it will avoid the coast to the left or to the right when it is encountered. That direction is reverted depending on whether the turtle is in pre‐ or postnesting migration (Figure 3).

Grid cells are characterized by their location. They can be of four different types: terrestrial, nesting site, feeding patch, or just ocean. Terrestrial cells are barriers to movement. Nesting sites represent main regional rookeries (Figure 2; Table 2). They are dispersed across the region with a higher concentration in the northwest of the map (north of the Mozambique Channel). Feeding patches, derived from telemetry mapping (Figure 2), are characterized by their resource level reflecting the availability of seagrass, the main forage for green sea turtles. The resource level of each feeding patch is constantly updated (growth or depletion) depending on the number of turtles feeding on it. Most of the feeding patches occur in larger clusters along continental shelves.

Under one simulation scenario, turtle movement is affected by oceanic currents derived from climatology maps: The turtle's velocity vector is resulting from the turtle's motor velocity vector plus the oceanic current velocity vector at turtle location. Ocean currents are represented via color coding of oceanic grid cells, in the RGB (red, green, blue) tuple: The red and blue components were used to represent, respectively, the eastward and the northward components of the sea surface currents. Feeding patches are possibly exposed to perturbations that alter their productivity. Perturbations are represented by a latitude coordinate and a spatial range of action. The growth rates of feeding patches located within the perturbations' spatial range are diminished with the amount of reduction depending on the feeding patch's distance to the perturbation's latitude.

Each simulation lasts for approximately 50 years (36,500 time steps). The first two years (1,500 times steps) are considered as a burn‐in period where no model output is recorded. Grid cell dimension is approximately 7 × 7 km; the entire model world consists of 567 × 577 grid cells, corresponding to 3,969 × 4,039 km.

#### 2.2.3 Process overview and scheduling

At each time step, which corresponds to half a day, first all green turtles and then all feeding patches are processed, both in randomized order and with immediate updating of their state variables. In the following, the names of submodels, which are described in detail in the ODD element “Submodels,” are given in parentheses.

The task a green turtle has to perform depends on its internal state: If the internal state is “feeding,” it feeds (win‐energy) and then possibly switches its internal state to “foraging‐migration” (foraging‐migration‐start) which includes selecting another feeding patch (allocate‐new‐feeding‐patch), or possibly switches to “prenesting” (prenesting‐migration‐start); if the internal state is “prenesting,” the turtle moves toward the nesting site (move‐one‐step‐toward) if it is still outside the detection range of the nesting site, otherwise the internal state switches to “nesting”; if the internal state is “postnesting,” it moves toward its current preferred feeding patch (move‐one‐step‐toward) if it is still outside the detection range of the feeding patch, otherwise the state switches to “feeding”; if the internal state is “nesting,” the turtle nests (nests), which includes a possible switch to the state “postnesting”; if the internal state is “foraging‐migration,” the turtle moves between feeding patches in the same way it moves on its way toward and back from its nesting site (move‐one‐step‐toward).

**Figure 4:**
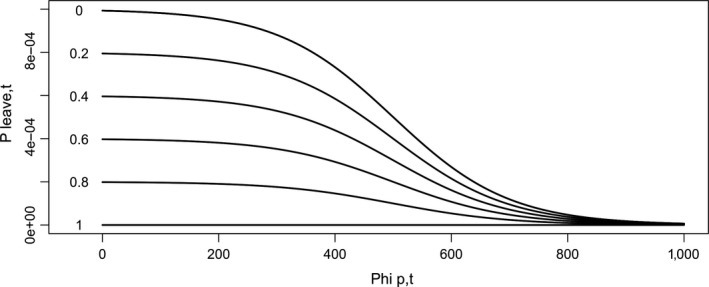
Foraging patch fidelity strategies and their functional relationships. This figure illustrates the probability *P*_leave,_ *_t_* for a turtle *i* to leave a patch *p* depending on its foraging patch fidelity strategy *S_F_*_,_ *_t_* and patch resource level Φ*_p_*_,_ *_t_*. The *x*‐axis represents the resource level Φ*_p_*_,_ *_t_* of the patch *p*. The *y*‐axis is the level of probability *P*_leave,_ *_t_* of leaving the patch at time *t*. Each curve depicts the probability *P*_leave,_ *_t_* of leaving the patch depending on actual level of patch resource. Turtle foraging fidelity patch strategy *S_F_*_,_ *_t_* is fixed across a single simulation. A foraging patch fidelity strategy closed to 0 (higher curves) leads to an overall higher probability to leave the patch (mover strategy). A strategy closed to 1 (lower curves) leads to an overall smaller probability of leaving the patch (stayer strategy)

At each time step, the turtles' energy level is updated by either gaining energy while feeding or losing energy while nesting or migrating. Individual actions rely on two decision strategies: foraging strategy and nesting allocation strategy. The foraging strategy specifies whether and when a turtle leaves its feeding patch for another one depending on the resource level of the actual feeding patch. The nesting strategy controls the amount of internal energy invested at each nesting event. We modeled the range of possible strategies in both processes, by a single index ranging from 0 to 1. A foraging patch fidelity strategy close to 1 leads to a “stayer strategy” while a foraging patch fidelity strategy *S_F_* close to 0 leads to a “mover strategy” (Figure 4). A nesting strategy close to 1 leads to an “investment strategy” while a nesting strategy close to 0 leads to a “conservative strategy.” We ran sets of simulations with various combinations of foraging and nesting strategies.

**Figure 5:**
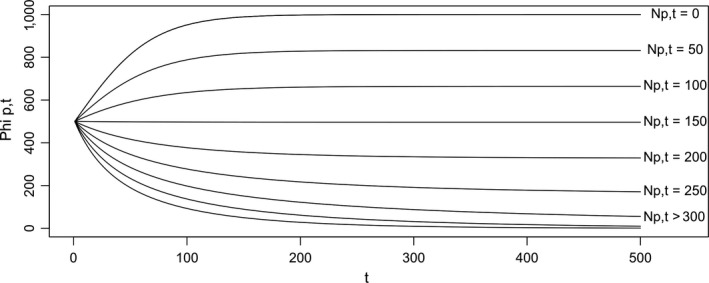
Temporal development of patch resource level Φ*_p_*_,_ *_t_* as a function of time *t* and number of sea turtle feeding on patch *N_p_*. The *y*‐axis represents the resource level Φ*_p_*_,_ *_t_* of the patch *p*. The *x*‐axis represents the time *t*. Each curve describes how the resource level Φ*_p_*_,_ *_t_* evolves depending on the number of turtles *N_p_*. During simulations, the resource level of a patch is not likely to evolve smoothly as suggested by these curves as the number of turtles' feeding on the patch may change between time steps

Movement is represented as direct movement toward a selected site, which is modified when barriers (islands, mainland) are encountered and possibly by passive drift due to oceanic currents. Movement is energetically costly, so that swimming between foraging patches or foraging further from the nesting site has to be balanced by a gain in foraging conditions. For the feeding patches, growth, depletion by turtles, and possibly perturbation of the amount of seagrass are considered (seagrass‐stock‐regrowth; Figure 5). Perturbation represents potential natural or anthropogenic impacts (e.g., climate change, habitat destruction, oil spill); its strength depends on latitude relative to the perturbation's location. Feeding patches that are not within the spatial range of action of the perturbation are not affected.

Finally, plots and file outputs are updated. Output analyses comprised spatial foraging and migrating pattern as well as reproductive output at the population scale in response to the turtle's strategies. It should be noted that the model did not include mortality or the turtles' life cycles; calculation of the population's reproductive output calculation was based on the number of nesting events and the energy individuals invested into eggs when nesting.

**Figure 6:**
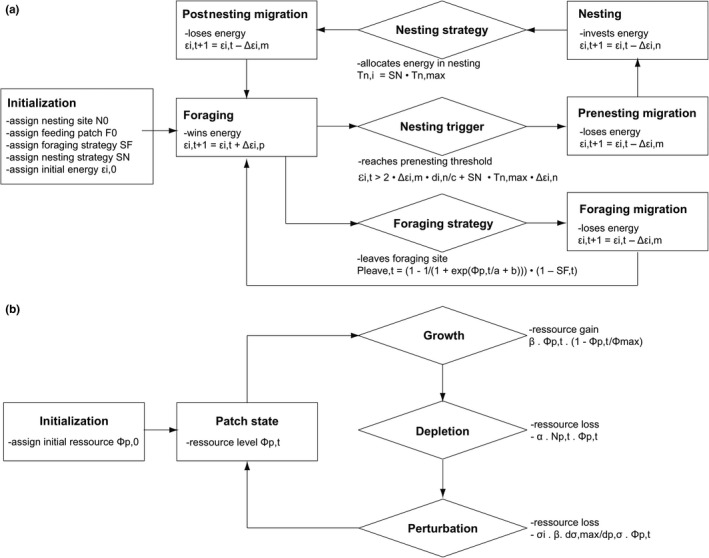
Flow chart of the model's processes. (a) Flow chart of the turtles' nesting‐migration‐foraging cycle showing the transitions between internal states. (b) Flowchart of the processed determining the resource level of feeding patches

**Figure 7:**
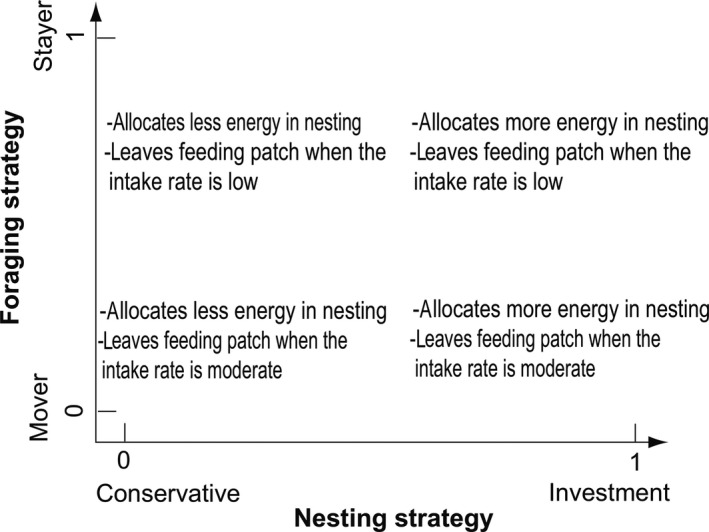
Categories of behavioral strategies. The *x*‐axis represents the nesting allocation strategy. The *y*‐axis represents the foraging patch fidelity strategy. Nesting and foraging strategy are constrained between 0 and 1. A nesting allocation strategy closed to 0 leads toward a “conservative” tendency and a nesting allocation strategy closed to 0 to an “investment” tendency. A foraging patch fidelity strategy closed to 0 tends toward a “mover strategy” and a foraging patch fidelity strategy closed to 1 toward a “stayer strategy”

Figure 6 summarizes the processes as implemented in the model. Figure 7 depicts the categories of behavioral strategies. Model parameters are specified in Table 1. When possible, the model was parameterized with field data. Otherwise, parameters were determined by inverse model fitting to the most realistic and biologically relevant observations.

**Table 1:** Model parameters and variables. Following values are expressed in daily units. Time step correction was taken into account directly in model implementation

(a) State variables			
Turtle	NetLogo variable	Abbreviation	Default value
Location	xcor, ycor	*x*, *y*	Variable
Preferred nesting site	gt‐nesting‐site	*N*_0_	1–14
Initial feeding patch	gt‐feeding‐patch	*F*_0_	1–47
Current feeding patch	gt‐feeding‐patch	*F_i_*	1–47
Internal state (prenesting, nesting, postnesting, foraging)	gt‐internal‐state	*–*	
Internal energy level at time step t	energy‐level	*ε_i_*_,_ *_t_*	Variable
Coast avoidance side (left or right)	gt‐avoidance‐side	*–*	−1 (left) or 1 (right)
Feeding patch			
Location	xcor, ycor	*p_x_*, *p_y_*	–
Feeding patch p resource level at time t	feeding‐patch‐resource‐level	Φ*_p_*_,_ *_t_*	Variable

(b) Parameters				
World	NetLogo variable	Abbreviation	Default value	
Number of turtles	*N*‐GTURTLES	*N_T_*	7,000	
Number of feeding patches	*N*‐FEEDING‐PATCHES	*N_F_*	47	
Number of nesting sites	*N*‐NESTING‐SITES	*N_N_*	14	
Perturbation latitude	perturbation‐latitude	*σ_y_*	−26°S	
Perturbation range	perturbation‐range	*d_σ_*_,max_	1,000 km	
Perturbation intensity	perturbation‐intensity	*σ_i_*	0.1	
Feeding patch allocation exponent	feeding‐patch‐allocation‐exponent	λ	20	
Turtle				
Foraging patch fidelity strategy	foraging‐fidelity‐strategy	*S_F_*		(0.2, 0.4, 0.6, 0.8)
Nesting allocation strategy	nesting‐allocation‐strategy	*S_N_*		(0.2, 0.4, 0.6, 0.8)
Prenesting threshold	prenesting‐threshold	ε_cycle_		Variable
Migration speed	migration‐speed	*c*		65 km.day^−1^
Energy loss per movement step during migration	energy‐loss‐migration	Δ*ε_i_*_,_ *_m_*	−1	
Energy loss per nesting day	energy‐loss‐nesting	Δ*ε_i_*_,_ *_n_*	−5	
Energy gain per time step while feeding	energy‐gain‐feeding	Δ*ε_i_*_,_ *_p_*_,_ *_t_*	Variable	
Proportion of intake from each patch	intake‐proportion	*α*	0.0001	
Distance to nesting site	distance‐from‐nesting‐site	*d_i_*_,_ *_n_*	Variable	
Maximum number of nesting duration	max‐nesting‐duration	*T_n_*_,max_	45 days	
Feeding patch				
Maximum feeding patch resource level	maximum‐feeding‐patch‐resource‐level	Φ_max_	1,000	
Initial feeding patch resource level	feeding‐patch‐resource‐level	Φ_0_	[0–1,000]	
Regrowth rate of feeding patch	patch‐regrowth‐rate	*β*	*2 · α · N_T_*/*N_F_*	
Slope of reaction toward patch leaving decision	feeding‐patch‐leaving‐slope	*a*	100	
Threshold of reaction toward patch resource depletion level *ε_p_*	feeding‐patch‐leaving‐threshold	*b*	500	
Latitudinal distance to perturbation	distance‐to‐perturbation	*d_p_*_,_ *_σ_*	Variable	

#### 2.2.4 Design concepts

##### Basic principles

We assume that turtles have a spatial memory of their preferred feeding patch and their nesting site. A basic energy budget of energy gains during feeding and losses during migration and egg production determines migration patterns, reproductive output, and return intervals to the nesting sites. Preferred feeding patches will be left in the search of better patches if feeding efficiency falls below a certain threshold; this can happen because too many turtles are feeding on this patch or if regrowth of the forage, sea grass, is slow due to perturbations.

##### Emergence

Foraging (stayer or mover) or nesting (investment or conservative) strategies directly determine rookery reproductive output via individual behavior. Intuitively, the best individual strategy would be to feed on feeding patches close to the rookery, thus reducing the cost of migration. However, with conspecifics depleting the close patches, different strategies might be beneficial. The rookeries' reproductive outputs consequently emerged from individual behavior while searching for patches and deciding on nesting energy allocation. Furthermore, the time interval between every breeding event emerged from energetic constraints, as well as the distribution of the spatial feeding patch usage that we could compare with tracking data from field surveys.

##### Sensing

At any time step, a migrating turtle could assess the direction of the migration target (its feeding patch or its nesting site) and has the ability to head toward it. In addition, a turtle could sense and avoid any coastal area located within 100 km of its actual location. Turtles did not have the ability to sense or anticipate the oceanic currents. Turtles perceived the resource level of the feeding patch where they were feeding on. The decision to leave the feeding patch was taken in response to this level.

##### Interaction

There was no direct interaction between individuals in the model. However, indirect interaction between individuals was included indirectly via resource competition at feeding patches.

##### Stochasticity

Initial feeding patches are assigned randomly according to decreasing exponential probability function of the distance to the nesting site. The initial spatial distribution of the turtle on feeding patches is therefore variable between simulations although it is impacted by the regional landscape. During the course of the simulation, foraging behavior also leads to temporal and spatial stochasticity. The decision of leaving a feeding patch for another is a probability function that relies on the foraging strategy and on the resource level of the feeding patch. Thus, individuals, although they share the same foraging strategy for a given simulation, will not leave the feeding patch simultaneously. Some individuals will randomly leave the patch earlier, therefore causing other individuals to remain in the patch. Furthermore, the choice of the new feeding patch is also a decreasing exponential function of the distance to the patch that is left. Turtles leaving a given patch will not travel to the same feeding patch affecting the occupation of the feeding patches.

The stochasticity here is implemented to reflect sources of variations that may actually occur during foraging phases. Stochasticity in turtle's distribution over the feeding patches will affect spatial usage of the oceanic areas as migratory corridors but also reproductive output of nesting sites. Over numerous simulations, we may identify areas that are of interest for feeding or migration, despite possible sources of random variations in spatial behavior. On the other hand, we may also identify robust tendencies in reproductive output variations between rookeries.

##### Observation

Focusing on model purposes, model outcomes comprised spatial foraging and migration pattern as well as reproductive output at the population scale in response to the turtle's strategies. To study foraging and migration patterns, we respectively measured feeding patch usage and mapped corresponding migration pathways. For this, we pooled for each environmental scenario the results from all combinations of the two behavioral strategies. We further observed the remigration interval as well as energy storage from which we deduced a reproductive output at rookeries. This was done separately for each behavioral strategy.

We studied spatial patterns of three foraging statistics: (a) time usage, that is, the sum, over all time steps, of the number of turtles present on a feeding patch at each time step, (b) number of postnesting visits, that is, the number of times that a turtle arrived in a feeding patch following postnesting migration, and (c) number of foraging visits, that is, the number of times that a turtle arrived in a feeding patch following foraging migration.

In addition, we also studied the foraging patterns in relation to the preferred nesting sites of the foraging turtles. For this, we computed two additional metrics: (a) the number of nesting sites from which nesters originated in a given feeding patch and (b) the diversity index of nesting sites from which nesters originated in a given feeding patch. Diversity index calculation *H_P_* is derived from Shannon's diversity index based on the number of postnesting visits:

(1) $$H_{p}=\frac{\Sigma_{N_{N}}r_{p,n}\times \ln\left(r_{p,n}\right)}{\ln\left(N_{N}\right)}$$

with *r_p_*_,_ *_n_* is the relative proportion of postnesting visits of patch *p* by turtles from nesting site *n*, and *N_N_* is the number of nesting sites present in the model.

Turtle's prenesting and postnesting migrations were recorded by randomly sampling individual's locations approximately every 500 time steps. Foraging migrations were not recorded. Migration pathways were then studied using kernel methods for density estimation on sampled locations (Worton, 1995).

Only the six main nesting sites (Europa, Aldabra, Mayotte, Mohéli, Tromelin, Glorieuses; see Table 2 for corresponding references) were considered in the study of the reproductive parameters. For each nesting site, the three following statistics were computed: (a) the mean individual remigration interval defined as the mean duration between successive nesting phases per each individual (Figure S2); (b) the mean individual energy level at nesting defined as the mean energy level of turtles after the nesting event; (c) the rookery overall reproductive output which was calculated as a function of the number of nests, the remigration intervals, and the energy level at nesting.

**Table 2:** Estimated number of nesting females for each nesting sites as available in the literature and relative proportion of nesting females assigned to each nesting site in the model. Absolute number of females actually assigned to each nesting site in the model was calculated, in respect of the relative proportion indicated here, ensuring that the total number of individuals in the model equals NT (7,000 individuals). This data need to be taken with caution and need to be justified by the papers cited. Comparisons need to be done with caution because estimation methods vary for all sites. (*Major nesting sites)

Site	Trigram	Area of estimation	Estimated number of females per year	Sources	Adjusted number of females per year	Relative proportion of females assigned in the model
Europa*	EUR	All	6,000–11,000	Le Gall, Bosc, Château, and Taquet (1986)	11,000	10,000
Aldabra*	ALD	All	3,000–5,000	Mortimer, Brandis, et al. (2011b)	5,000	5,000
Mayotte*	MAY	All	3,000–5,000	Bourjea, Frappier, et al. (2007a)	5,000	5,000
Mohéli*	MOH	6 beaches	4,410	Bourjea, Dalleau, et al. (2015a)	5,000	5,000
Tromelin*	TRO	All	1,430	Lauret‐Stepler et al. (2007)	2,000	2,000
Glorieuses*	GLO	60%	1,480	Lauret‐Stepler et al. (2007)	2,000	2,000
Tanzania	TAN	All	120–150	Muir (2005)	150	200
Iranja	IRA	All	100–150	Bourjea et al. (2006)	150	200
Juan de Nova	JUA	All	<80	Lauret‐Stepler, Ciccione, and Bourjea (2010)	70	200
Seychelles (Except Aldabra)	SEY	–	13–24	Mortimer, Camille, et al. (2011a)	150	200
Mozambique (Vamizi Island)	VAM	85%	50	Garnier et al. (2012)	60	200
La Réunion	RUN	All	<5	Ciccione and Bourjea (2006)	5	200
Kenya	KEN	–	Unknown	Okemwa et al. (2004)	Unknown	200
Mauritius (Chagos Archipelago)	CHA	–	–	–	–	200

To compute these statistics, at each time *k* a turtle *i* nested at nesting site *n*, we recorded the date *T_i_*_,_ *_k_* and the corresponding energy level after nesting *ε_i_*_,_ *_k_*. We computed the remigration interval as the time difference since the previous nesting event, *T_i_*_,_ *_k_*_,_ − *T_i_*_,_ *_k_*_−_ *_1_*. We computed the overall reproductive output RO*_n_* of each nesting site *n* as directly proportional to the energy levels at nesting *ε_i_*_,_ *_k_* and the nesting investment *S_N_*:

(2) $${\text{RO}}_{n}=\Sigma_{i}\Sigma_{k}\varepsilon_{i,k}/\left(T_{i,k}-T_{i,k-1}\right)S_{N}$$

#### 2.2.5 Initialization

The landscape, in particular the number and location of nesting and feeding patches, remained identical within and between simulations and was taken from input maps. Initial resource level of the feeding patch was either set to a random positive value sampled from a uniform distribution between zero and maximum resource level Φ_max_ or, if no depletion by turtles was considered, to Φ_max_.

Most simulations were run with 7,000 turtles. At the beginning of each simulation, the turtles' nesting sites were allocated randomly with the constraint of ensuring that realistic proportions were distributed over the nesting sites; that is, the distribution across nesting sites follows the known size of the nesting population (Table 2). The initial feeding patch was also assigned randomly assuming that the probability of a feeding patch to be assigned to a turtle is inversely proportional to the distance separating this site from the turtle's nesting site. That is, the initial distribution over across feeding patches follows an inverse exponential distance from the nesting site. This probability was calculated in the same way as the choice of a new feeding patch during foraging (procedure “allocates‐new‐feeding‐patch”). The preferred feeding patch may change during simulations depending on its quality.

At initialization, all turtles have the internal state “feeding” and are released at the location of their feeding patch. The initial internal energy level *ε*_0_ is randomly attributed by sampling from a positive uniform distribution between 0 and the total energy required for a whole nesting cycle.

#### 2.2.6 Input data

Main inputs for the model are the functional habitat map (rookeries maps for nesting sites and seagrasses for feeding patches) and the map of oceanic currents.

##### Rookeries

Rookery locations are mapped from local knowledge and using the latest available estimates of the number of annual nesting females (respective studies used are cited in Table 2). We are using the upper limit field estimation of nesting female's number to compute the proportion of individuals associated with each rookery in the model. The proportion of individuals assigned to each rookery is shown in Table 2. A minimum of 45 turtles is allocated to the smallest rookeries.

##### Feeding patches

Locations of feeding patches were set up by combining maps from two distinct sources: the World Atlas of Seagrasses (Green & Short, 2003) and the Agulhas and Somali Current Large Marine Ecosystem project (ASCLME; http://www.asclme.org). Mapped seagrass beds were transformed into feeding patches (grid cells) at locations corresponding to the location of the main mapped sea grasses beds. Additional feeding patches were added along the coast of Somalia as this place is known to host vast areas of seagrass bed that are not mapped in the cited datasets (S. Andréfouët, personal communication).

##### Oceanic currents

To model oceanic currents, we are using an annual climatology map that reflects the mean current velocities in the region. This map was computed by combining GEKCO surface current daily datasets (Sudre, Maes, & Garçon, 2013). We did not consider any seasonal effect at this stage. To represent the 2D currents vector maps in the model, in the RGB (red, green, blue) tuple that is used to encode colors in NetLogo, the green component was left at zero and the values of the red and blue component were used to represent, respectively, the eastward and the northward components of the sea surface currents (Figure 2).

#### 2.2.7 Submodels

##### Win‐energy

When at time *t* turtle *i* feeds on patch *p*, its internal energy level *ε_i_*_,_ *_t_* is increased:

(3) $$E_{i,t+1}=\varepsilon_{i,t}+\Delta \varepsilon_{i,p,t}$$

with Δ*ε_i_*_,_ *_p_*_,_ *_t_* being the net gain from patch *p* at time *t*. We do not explicitly consider metabolic costs for maintenance as this was assumed a constant variable independent from internal state. The net gain per time step Δ*ε_i_*_,_ *_p_*_,_ *_t_* depends on the resource level of the feeding patch Φ*_p_*_,_ *_t_*:

(4) $$\Delta \varepsilon_{i,p,t}=\alpha \cdot \Phi_{p,t}$$

with *α* being the depletion coefficient.

##### Foraging‐migration‐start

The probability *P*_leave_,*_i_* for turtle *i* to leave the actual feeding patch for another one depends on the resource level of the actual patch Φ*_p_*_,_ *_t_* and on its own foraging patch fidelity strategy *S_F_*. The functional relationship was modeled with a logistic curve:

(5) $$P_{\text{leave,}i}=\left(1-1/\left(1+\text{exp}\left(\left(\Phi_{p,t}+b\right)/a\right)\right)\right)/1000\cdot \left(1-S_{F}\right)$$

where *a* modulates the steepness of the reaction and *b* is the leaving threshold. A foraging patch fidelity strategy *S_F_* close to 1 leads to a “stayer strategy.” A foraging patch fidelity strategy *S_F_* close to 0 leads to a “mover strategy” (Figure 7). Values for parameters *a* and *b* are given in Table 1. The resulting probability of leaving a feeding patch depending on foraging strategy *S_F_* and feeding patch resource level Φ*_p_*_,_ *_t_* is illustrated in Figure 4. This submodel neither takes into account travel costs nor leaving the patch when its food intake drops below the average food intake on all other patches, since the energetic cost to another feeding patch that the turtle has never visited should be unknown to a turtle. Similarly, a turtle on a patch has no knowledge of the potential level of food intake it could get from other patches as it has to be located on a patch to know that level.

Therefore, the cost of foraging exploration will emerge from the model.

##### Allocate‐new‐feeding‐patch

The selection of a new feeding patch was distance‐dependent with selection probability *P*_selection_ determined by an exponential decay function:

(6) $$P_{\text{selection}}={\left(1-d_{\text{relative}}\right)}^{\lambda }$$

where *d*_relative_ *= d* − *d*_min_/*d*_max_ − *d*_min_ is calculated from *d*, the distance between a new feeding patch and the current feeding patch, and *d*_min_ and *d*_max_, the minimum and maximum distance between feeding patches. *λ* is an arbitrary exponential decay coefficient. This model assumes that choice of a new feeding patch is based rather on the turtles' better knowledge of the location of feeding patches nearby than by those feeding patch resource levels, which they cannot know. The minimum and maximum possible distances are not known to the turtles but used to scale the spatial scale of knowledge.

##### Move‐one‐step‐toward‐with/without‐currents

At each time step and for each turtle in migration, spatial location was updated with a fixed speed of 2.7 km/hr (65 km/day) and a heading toward the selected patch when not facing the coast. Speed value was derived from in situ satellite tracking measurement (Dalleau, 2013). Effective traveling speed and direction may, however, be impacted by oceanic currents at the turtle's location.

During prenesting, postnesting or foraging migration, at each time step *t* a turtle *i* moves toward a selected patch *p*, it loses a fixed amount of energy Δ*ε_i_*_,_ *_m_* (Table 1):

(7) $$\varepsilon_{i,t+1}=\varepsilon_{i,t}-\Delta \varepsilon_{i,m}$$

At each time step *t*, a turtle *i* attempts to move one step in the direction of the target, which is either its nesting site in the case of prenesting migration or its current preferred feeding patch in the case of postnesting or foraging‐migration.

For avoidance of coastal grounds, we implemented a simple wall‐following algorithm (Figure 3). At a given time step, if moving a turtle forward causes this turtle to encounter a coastal grid cell (patch‐ahead‐is‐coast?), its swimming direction is modified incrementally (angle‐step) up to the minimum angle that allows to move forward without encountering a terrestrial grid cell (see next paragraph regarding the direction of rotation). The turtle then moves forward. At the following time step, if possible, the swimming direction is first modified incrementally (angle‐step) to a direction closer to the direction of the target (the feeding patch or the nesting site) that allows moving forward without encountering a grid cell. If the direction of the target can be reached, the swimming direction of the turtle is set to the target's direction. Contrarily, if the swimming direction cannot be modified and if the turtle cannot moves forward, then the swimming direction is once again modified incrementally (angle‐step) by the minimum angle that allows to move forward without encountering a terrestrial grid cell. At the next time step, the same process is repeated. This algorithm leads the turtle to follow the coast until it can freely move in the direction of the target once again.

Regarding the rotation direction (to the left or to the right), the first time that a turtle encounters a coast, it corresponds to the direction that leads to the least turning angle required to avoid the coast. The rotation direction is then memorized (gt‐avoidance‐rotation‐direction) and will remain the same during the duration of a given migration. Nevertheless, the rotation direction is reverted when a turtle starts a pre‐ or a postnesting migration. This reversion is implemented to favor, at least partially, symmetrical migration trajectories between pre‐ and postnesting migration (Figure 3). With that we ensure that an equivalent route is followed on the way to and the way back from the nesting site. In other terms, if the turtle followed the coast to the right going to the nesting site, it will follow it to the left on the way back. Additionally, the rotation direction is also reset each time that a turtle starts and stops a foraging migration since these migrations are independent from nesting migrations and since they modify the current feeding patch of the turtle.

In case the effect of oceanic currents on movement is considered, migration direction is modified according to the oceanic current velocity at actual turtle position. The final velocity vector is resulting from the turtle's motor velocity vector toward the target plus the oceanic current velocity vector at turtle location. Computationally, this is simply implemented by artificially displacing the target site (feeding patch or nesting site) at each time step. The “artificial” target site (*x*′, *y*′) is located at the location of the turtle (*x*, *y*) to which we added the vector sum of the velocity vector in the absence of current (d*x*, d*y*) and the current velocity vectors (*xc*, *yc*). It was calculated as follows:

(8a) $$x^{\prime }=x+dx+xc$$

(8b) $$y^{\prime }=y+dy+yc$$

The algorithms to move one step forward and to avoid the coastal grounds are then similar than in the absence of currents.

##### Prenesting‐migration‐start

The decision to start prenesting migration depends on the estimated level of energy necessary to complete the entire nesting process, that is, the turtles stop feeding only if they gained a sufficient amount of energy to complete a round‐trip migration to the nesting site and nesting action. A turtle therefore starts prenesting migration (from its current feeding patch to its nesting site) when its energy level *ε_i_*_,_ *_t_* reaches approximately the total energy level needed to complete the cycle, *ε*_cycle_:

(9) $$\varepsilon_{\text{cycle}}=2\cdot \varepsilon_{\text{migration}}+\varepsilon_{\text{nesting}}=2\cdot \Delta \varepsilon_{i,m}\cdot d_{i,n}/c+S_{N}\cdot T_{n,\text{max}}\cdot \Delta \varepsilon_{i,n}$$

where Δ*ε_i_*_,_ *_m_* is the energy lost on each time during migration, *d_i_*_,_ *_n_* the distance from the current feeding patch to the nesting site, and *c* migration velocity.

##### Nests

Depending on the nesting strategy considered, an individual could either invest a large amount of energy into nesting (“investment strategy”—the big spender), thereby trading off between high nesting investment and low nesting frequency (Figure 7). This might possibly result in large intervals between nesting, thereby reducing fitness when considered over lifetime average. Alternatively, an individual could invest only a limited fraction of energy for nesting (“conservative strategy”—bank saver), thereby reducing the nesting investment with lower numbers of eggs produced but shortening the interval between nesting phases.

The number of time steps spent at nesting sites depends on the value of the parameter characterizing the nesting strategy *S_N_*:

(10) $$T_{n,i}=S_{N}\cdot T_{n,\text{max}}$$

During nesting, at each time step *t* spent at a nesting site *i*, an individual loses Δ*ε_i_*_,_ *_n_*:

(11) $$\varepsilon_{i,t+1}=\varepsilon_{i,t}+\Delta \varepsilon_{i,n}$$

A nesting strategy *S_N_* close to 1 leads to an “investment strategy.” A nesting strategy *S_N_* close to 0 leads to a “conservative strategy.” After completing the nesting event, the turtle goes back to its last preferred feeding patch.

##### Seagrass‐stock‐regrowth

We considered regrowth of seagrass feeding patches based on a logistic function (Figure 5). Uptake resources by turtles was density‐dependent (see Bjorndal, Bolten, & Chaloupka, 2000, e.g., of in situ density‐dependence); that is the individual uptake per time step decreased as the number of turtles actually foraging on the patch increased. Depending on its foraging strategy, a turtle could tolerate a low patch resource level and avoid costly foraging migration (“stayer” tendency) or could rather leave a feeding patch when its resource level is too low (“mover” tendency). At each time step *t*, the resource level Φ*_p_*_,_ *_t_* of the feeding patch *p* is updated:

(12a) $$\Phi_{p,t+1}=\Phi_{p,t}+\Delta_{\Phi p,t}$$

where ΔΦ*_p_*_,_ *_t_* is the net growth of patch *p* at time *t* which depends on depletion by *N_p_*_,_ *_t_* turtles foraging on this patch at time *t* and regrowth according to a logistic growth model:

(12b) $$\Delta_{\Phi p,t}=\beta \Phi_{p,t}\left(1-\Phi_{p,t}/\Phi_{\text{max}}\right)-\alpha N_{p,t}\Phi_{p,t}$$

where *α* is the depletion coefficient. The coefficient *β* was adjusted to (a) maintain the amount of resources relatively constant across the simulation; (b) make the long‐term average resource level being about half of the maximum resource level common to all feeding patches, this level was chosen arbitrarily but was shared across all simulations; and (c) assuming that the turtles are evenly distributed over the feeding patches.

Mathematically, this means for all patches *p*:

(13a) $$\Delta_{\Phi p,t}\approx 0$$

(13b) $$\Phi \approx \Phi_{\text{max}}/2$$

(13c) $$N_{p,t}=N_{T}/N_{F}$$

which gives the following:

(14) $$\beta =\alpha \cdot N_{T}/N_{F}\cdot \Phi_{\text{max}}/\left(\Phi_{\text{max}}-\Phi_{\text{max}}/2\right)=2\cdot \alpha \cdot N_{T}/N_{F}$$

The development of the resource level Φ*_p_*_,_ *_t_* of a feeding patch depending on the number of turtles *N_p_*_,_ *_t_* foraging on it is illustrated in Figure 5.

##### Perturbation

Perturbation is defined by a latitude position *σ_y_*, an intensity level *σ_i_*, and a maximum range of action *d_σ_*_,max_. The impact of perturbation on a given feeding patch depends on its relative latitude *p_y_* to perturbation latitude *σ_y_*. Perturbation effect on feeding patch resource level is inversely proportional to the latitudinal distance *d_p_*_,_ *_σ_* from the perturbation latitude position *σ_y_* and is also depends on the regrowth rate of a feeding patch. At each time step, the patch resource level is perturbed as follow:

(15a) $$\text{if}\;d_{p,\sigma }<d_{\sigma ,\text{max}}:\Phi_{p,t+1}=\Phi_{p,t}-\Delta_{\Phi p,t}$$

That is, if the feeding patch is within the perturbation range (*d_p_*_,_ *_σ_* < *d_σ_*_,max_), the patch resource level for the next step (Φ*_p_*_,_ *_t_*_+1_) is diminished by a certain delta (ΔΦ*_p_*_,_ *_t_*). Equation 15b details how this delta is calculated: The diminishing delta is proportional to the perturbation intensity (*σ_i_*), the relative latitude to the perturbation location (*d_σ_*_,max_/*d_p_*_,_ *_σ_*). It is also a fraction of the actual patch resource level (Φ*_p_*_,_ *_t_*). The coefficient β is calculated to ensure a sufficient “global” energy level in the system (see previous paragraph Equation 14), with

(15b) $$\Delta_{\Phi p,t}=\sigma_{i}\cdot \;\beta \;\cdot \;d_{\sigma ,\text{max}}/d_{p,\sigma }\cdot \Phi_{p,t}$$

(15c) $$d_{p,\sigma }=p_{y}-\sigma_{y}$$

Equation 15c is correcting latitude effects and shows the relative latitude of the patch to the latitude of the perturbation. Note that this will remain positive as perturbation latitude is south of the southern site.

### 2.3 Simulation experiments

When possible, the model was parameterized with field data. Coastlines were simplified from GEBCO (General Bathymetric Chart of the Ocean) gridded global bathymetry data (http://www.gebco.net). Rookery locations are mapped using the latest available estimates of annual nesting female numbers (Table 2). Oceanic currents were derived from climatology maps (Sudre et al., 2013). Average swimming speed during migration (65 km/day) was derived from in situ satellite tracking measurement on female green turtles in the region (Dalleau, 2013).

Locations of feeding patches were set up by combining maps from two distinct sources: the World Atlas of Seagrasses (Green & Short, 2003) and the Agulhas and Somali Current Large Marine Ecosystem project (ASCLME; http://www.asclme.org). Mapped seagrass beds were transformed into feeding patches (grid cells) at locations corresponding to the location of the main mapped sea grasses beds. Additional feeding patches were added along the coast of Somalia as this place is known to host vast areas of seagrass bed that are not mapped in the cited datasets (S. Andréfouët, personal communication).

Otherwise, parameters were determined by inverse model fitting to the most realistic and biologically relevant observations. For our simple energy budget model, we assumed that sea turtles' reproductive activities are considerably more energetically costly than swimming or foraging (Williard, 2013). Here, cost of nesting compared to other activities was calibrated by aiming for a remigration interval in the model (time interval between individual nesting seasons) ranging between 2 and 7 years across all simulations. These values match the range observed worldwide (Troeng & Chaloupka, 2007). Seagrass growth and density‐dependent depletion were adjusted to maintain the amount of resources relatively constant across a simulation.

Each model simulation was run for approximately 50 years. The first two years were considered as a burn‐in period where no model output was recorded in order to avoid possible artifacts generated by the arbitrarily chosen initial state of the model entities. We ran simulations under three environmental scenarios: scenario 1, without oceanic currents; scenario 2, with oceanic currents; and scenario 3, without oceanic currents but with local perturbations (i.e., selective reduction of feeding sites' productivity). Under environmental scenario 3, perturbations were only located in the southern feeding patches. We arbitrarily chose a single location to simplify our understanding of the effect of the perturbation in the model. Please note that we do not consider a model without oceanic currents (i.e., scenario 1) as realistic but wanted to assess their effects. Exploring unrealistic scenarios is an important element of model analysis and has been listed as part of “Robustness Analysis” (Grimm & Berger, 2016). For each scenario, we ran five repetitions for combinations of different nesting and foraging strategies, respectively, that is, conservative/investment tendencies and mover/stayer tendencies (Figure 7). Strategy tendencies were fixed and equal for all turtles throughout a single simulation. Overall, we ran a total of 240 simulations (Table 3).

**Table 3:** Model simulation experiments. Overall, we ran three scenarios, four foraging strategy tendencies, four nesting strategy tendencies, and five repetitions for each configuration leading to a total of 240 simulation runs

Scenario	Oceanic current	Perturbations	Foraging strategy	Nesting strategy	Repetitions	Simulations
Scenario 1	No	No	4	4	5	80
Scenario 2	Yes	No	4	4	5	80
Scenario 3	No	Yes	4	4	5	80

### 2.4 Observation and analysis of model output

Model outcomes comprised spatial foraging and migration pattern as well as reproductive output in response to the turtle's strategies. To study foraging and migration patterns, we respectively measured feeding patch usage and mapped corresponding migration pathways. We pooled, for each scenario, the results from all combinations of the behavioral strategies' tendencies. Therefore, we did not assess the spatial effects of behavioral strategies within a given scenario but rather between scenarios. However, emergent biological properties such as remigration interval, energy storage, and reproductive output at rookeries were analyzed in the light of behavioral strategies within each scenario.

#### 2.4.1 Feeding patch usage

We studied spatial patterns of three foraging statistics: (a) time usage, that is, the sum, over all time steps, of the number of turtles present on a feeding patch at each time step, (b) number of postnesting visits, that is, the number of times that a turtle arrived in a feeding patch following postnesting migration, and (c) number of foraging visits, that is, the number of times that a turtle arrived in a feeding patch following foraging migration from another feeding patch.

In addition, we also studied the foraging patterns in relation to the nesting sites of origin for the foraging turtles. For this, we computed two additional metrics: (a) the number of nesting sites from which nesters originated in a given feeding patch and (b) a diversity index of nesting sites from which turtles originated in a given feeding patch.

#### 2.4.2 Migration pathways

Turtle's prenesting and postnesting migrations were recorded by randomly sampling individual's locations approximately every 500 time steps. Foraging migrations were not recorded. Migration pathways were then studied using kernel methods for density estimation on sampled locations (Worton, 1995).

#### 2.4.3 Energy at nesting, remigration interval, and reproductive output

Only the six main and well known nesting sites (Europa, Aldabra, Mayotte, Mohéli, Tromelin, Glorieuses; Figure 2; Table 2) were considered in the study of the reproductive parameters. For each nesting site, the three following statistics were computed: (a) the mean individual remigration interval defined as the mean duration between successive nesting phases per each individual; (b) the mean individual energy level at nesting defined as the mean energy level of turtles after the nesting event; (c) the rookery overall reproductive output which was calculated as the sum over each individual's nesting event, that is, the sum of the energy‐level ratio of each nesting turtle by the remigration interval.

## 3 RESULTS

### 3.1 Feeding patch usage

**Figure 8:**
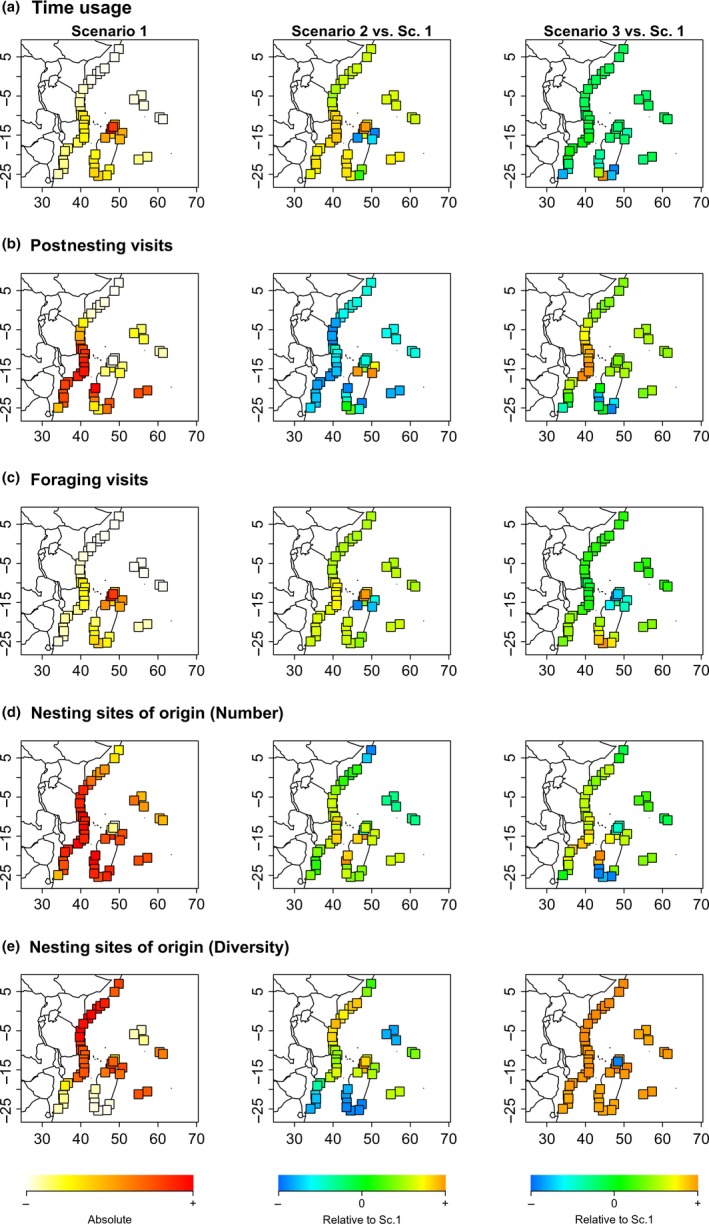
Usage of feeding patches. Left panels describe usage of feeding patches under scenario 1, center and right panel, respectively, describe feeding patch usage under scenario 2 and scenario 3 relative to scenario 1. Rows of panels correspond to different usage statistics: (a) time usage, that is, total number of time steps spent by turtles on this site; (b) number of postnesting visits, that is, number of times that a turtle reached this site following a postnesting migration, (c) number of foraging visits, that is, number of times that a turtle reached this site following a foraging migration, (d) number of sites from which foraging turtles originate, (e) diversity (Shannon index) of sites from which foraging turtles originate; this index reflects the “proportion” of nesting sites from which foraging turtle originate. All statistics are calculated over the all sets of simulation for each scenario, that is, 5 simulations for each of the 4 × 4 combinations of foraging and nesting strategies taken in (0.2, 0.4, 0.6, 0.8; 80 simulations per scenario)

Under environmental scenario 1 (without oceanic currents nor perturbations), the most frequented feeding patches were located on the coasts of Madagascar, Mozambique, and Tanzania. These regions had higher levels of usage in terms of time usage, postnesting visits, and foraging visits (Figure 8a–c, left panels). The northwestern part of Madagascar appeared as one of the most important foraging regions as the feeding patches located in this area showed the highest levels of time usage (Figure 8a, left panel). Least visited areas corresponded to the eastern island sites (Mascarene and Seychelles) and the extreme northern sites located along the Somali coast. Regarding the nesting sites of origin, high levels of mixing were observed throughout the region (Figure 8d, left panel). Feeding patches located in the south of the Mozambique Channel had low values for the diversity index of nesting sites, that is, turtles' feeding in these patches originated only from few nesting sites (Figure 8e, left panel). Feeding patches along the Somali coast, despite low usage, showed high diversity levels in the nesting sites of origin.

Under environmental scenario 2 (including oceanic currents), in comparison with scenario 1 (without oceanic currents), main differences in time usage and number of visits occurred in the eastern coast of Madagascar (Figure 8a–c, center panels). When considering the currents, the feeding patches located in this area exhibited lower levels of time usage and of foraging visits (Figure 8a,c, center panels) but higher numbers of postnesting (Figure 8b, center panel). Regarding nesting sites of origin, sea currents increased the variability in the diversity patterns (Figure 8d,e, center panels). In this case, patches located at the edges of the region, such as the northern and southern sites of the east African coast as well as the isolated islands, were visited by turtles from a smaller number of nesting sites (Figure 8d,e, center panel).

Under environmental scenario 3 (with perturbations), southern feeding patches (ca. 15°S to 25°S of Latitude) were exposed to perturbations. In comparison with scenario 1, it induced lower levels of postnesting visits in the southern patches (Figure 8b, right panel) and higher levels of foraging migrations (Figure 8c, right panel). Nevertheless a few southern patches in the southwest of Madagascar had exceptionally high levels of time usage. As another consequence of perturbations, pressure on the northern patches was increased as they were more frequently visited (Figure 8b, right panel).

### 3.2 Migration patterns

**Figure 9:**
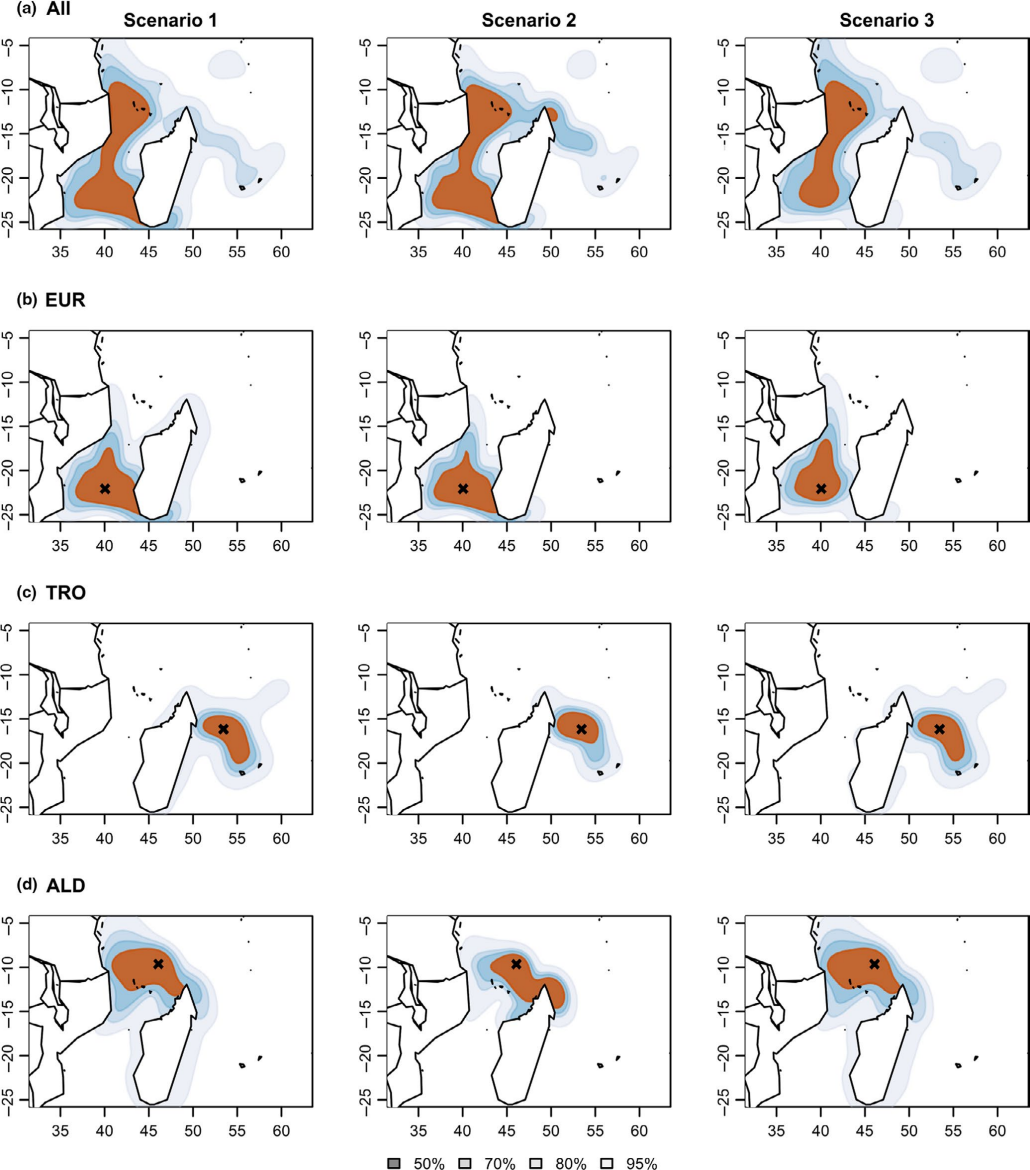
Kernel densities of migration pathways under the three scenarios. Densities for scenario 1 (left panel), scenario 2 (center panel), and scenario 3 (right panel) for (a) all individuals; individuals nesting in (b) Europa, (c) Tromelin, and (d) Aldabra. (b, c, d) The nesting island is represented with the black cross. Kernels were calculated over the all sets of simulations for each scenario, that is, 5 simulations for each of the 4x4 combinations of foraging and nesting strategies taken in (0.2, 0.4, 0.6, 0.8), by random sampling 125 positions per simulation (10,000 positions per scenario)

Kernel density analysis (Figure 9a, left panel) showed important postnesting migratory areas around the islands of the northern part of the Mozambique Channel as well as around the island of Europa, south of the Channel. Two migration corridors were observed (a) a major trident shaped corridor, between the northern coast of Mozambique, the southern coast of Mozambique, and the southern coast of Madagascar; (b) and another important one between the northern coast of Mozambique to the Comoros Archipelago. Adding oceanic currents (scenario 2, with oceanic currents, Figure 9a, center panel) mostly modified the migratory dynamics within the northern part of the Mozambique Channel. The migration corridor of this area was broadened to the northern coast of Madagascar and beyond to the small nesting island of Tromelin. Under scenario 3 (Figure 9a, right panel), the main effect of perturbation in the southern patches affected the trident shaped corridor, with a loss of movements between the southern coast of Mozambique and the southern coast of Madagascar.

When looking at analyses for particular nesting sites (Figure 9b–d), under scenario 2 after nesting, individuals from Tromelin migrated more frequently with the main current flow, the south equatorial current (SEC), preferably toward the northwest of Madagascar than the Mascarene islands (Reunion and Mauritius). Individuals from Aldabra also migrated preferably along the North‐Equatorial Madagascar current (NEMC) flow (8d, center panel). Similar patterns were observed for the other nesting islands of the northern part of the Mozambique Channel: Glorieuses, Mayotte, and Mohéli (results not shown here). Under scenario 3 (perturbed foraging sites), individuals nesting on Europa avoided migration toward perturbed feeding patches, in the south of the Mozambique Channel (Figure 9b, right panel), and they preferred migration along the Europa‐North Mozambique axis, which reinforced the importance of this major migration corridor.

### 3.3 Reproductive output across the region

**Figure 10:**
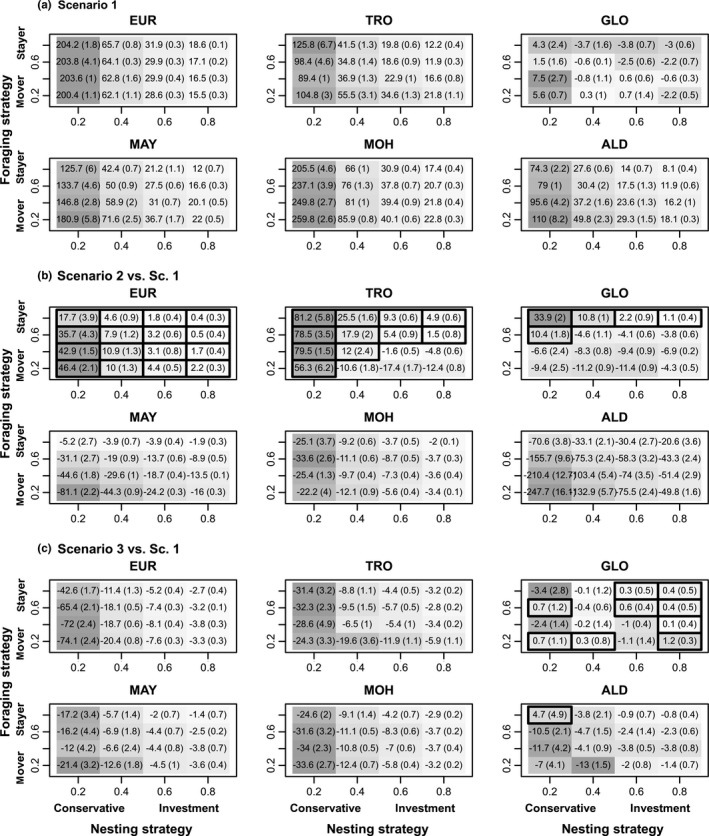
Reproductive output for the six main nesting sites. Reproductive output as a function of nesting allocation strategy (*x*‐axis) and foraging patch fidelity strategy (*y*‐axis). Boxed values are positive. (a) Reproductive outputs for scenario 1, (b) reproductive outputs for scenario 2 relative to Scenario 1, and (c) reproductive outputs for scenario 3 relative to scenario 1. (ALD, Aldabra; EUR, Europa; GLO, Glorieuses; MAY, Mayotte; MOH, Mohéli; TRO, Tromelin). Gray gradient indicates highest values (dark gray) to lowest values (light gray). A diagram representation of this figure is available in Figure S3

Under all scenarios, site‐specific results showed high spatial variability in reproductive output (Figure 10). Europa and Mohéli Islands had the highest level of reproductive output. Glorieuses archipelago had the lowest reproductive output. Impact of oceanic currents (Figure 10b) had contrasting influence across the region. For a majority of sites (Mayotte, Mohéli, Aldabra) oceanic currents lowered the reproductive output, sometimes drastically (e.g., in the case “mover” and “conservative” strategies in Aldabra, Figure 10b). Yet, for Europa Island, oceanic currents had positive impacts on reproductive output regardless of the decisions strategies (Figure 10b). For Glorieuses, only “stayer” tendencies led to superior reproductive output. The patterns were similar but combinations of “mover” and “conservative” tendencies also led to higher reproductive output. Perturbations of southern feeding patches (Figure 10c) had a negative impact on reproductive output, especially for Europa Island, the nearest site from the perturbed patches, and particularly for “mover” and “conservative” tendencies. Reproductive output of all nesting sites was affected regardless of the decision strategies (with the exception of Glorieuses).

### 3.4 Reproductive output under behavioral strategies

Detailed results regarding energy at nesting and remigration intervals (duration between two nesting phases) are presented in the Supplement. Reproductive output was maximal under scenario 1 (Figure 10a). Oceanic currents (scenario 2, Figure 10b) or human perturbations (scenario 3, Figure 10c) both had overall negative impact on reproductive output. Nevertheless, in some rare cases for “conservative” nesting tendencies mean reproductive output was higher when considering ocean currents (Figure 10b, left panel). Reproductive output under scenario 1 was lower for “investment” nesting tendencies than for “conservative” nesting tendencies. To a lesser extent, it was also slightly decreasing for “stayer” foraging tendencies. Nesting strategy did not have any influence on the trend in reproductive output when considering ocean currents (Figure 10b, left panel). However, when introducing perturbations the loss in reproductive output was more pronounced in “conservative” tendencies than “investment” nesting tendencies (Figure 10c, left panel). On the other hand, while foraging strategies had little impact on reproductive output under perturbation scenario (9c, right panel), they modified reproductive output in response to oceanic currents. Here, we observed a higher loss in reproductive output for “movers” than “stayers.” To summarize, the model predicted that “stayer” foraging tendencies should perform better when migration was strongly affected by oceanic currents while in perturbed environments “investment” nesting tendencies limited the loss in reproductive output.

## 4 DISCUSSION

### 4.1 The importance of landscape configuration on the spatial ecology of sea turtles

#### 4.1.1 Landscape configuration spatially structures sea turtles' populations

Migratory corridors and foraging hot spots are commonly observed for green turtle populations worldwide (Luschi, Hays, Del Seppia, Marsh, & Papi, 1998; Read et al., 2014; Stokes et al., 2015; Troëng, Evans, Harrison, & Lagueux, 2005). By implementing simple movement and behavioral decision rules, we were able to reproduce the main regional patterns observed through genetic and tracking studies, as we discuss in detail below. Our results suggest that the spatial distribution of migration corridors and foraging hot spots is constrained by the intrinsic landscape configuration, that is, the relative location of nesting sites and foraging areas, land barriers, and oceanic currents. The initial choice of a foraging site for sea turtles might involve mechanisms more complicated than those implemented here, such as drifting pattern and imprinting during early life stages (Scott, Marsh, & Hays, 2014). Nevertheless, the model demonstrated that constraints occurring at the adult stage could explain and maintain observed spatial patterns in the field (migration corridors, foraging area usage, foraging area composition). The adult's environment, here in the shape of coastal barriers, oceanic currents, and perturbations, might modify migratory connectivity between sites.

#### 4.1.2 Landscape configuration affects reproductive output

Variations of reproductive output have been observed in various species of sea turtles through numerous parameters: remigration interval (i.e., breeding frequency), clutch frequency, clutch size, size, and nutritional components of eggs, hatching, and emergence success. Some parameters are affected by physiological constraints; for example, the clutch size is generally correlated with the size of the female (Broderick, Glen, Godley, & Hays, 2003; Hays & Speakman, 1991), or by local conditions at nesting sites; for example, the emergence success highly relies on incubation conditions (Mortimer, 1990). Variations in the parameters remigration interval and clutch frequency are mainly attributed to foraging and migration conditions (Broderick et al., 2003; Hatase, Omuta, & Tsukamoto, 2013; Hatase & Tsukamoto, 2008; Troeng & Chaloupka, 2007; Vander Zanden et al., 2014).

In the model, levels of reproductive output were very variable between rookeries under identical simulation parameters (Figure 10), suggesting that landscape structure affected reproductive potential in a way that similar behaviors led to various reproductive output depending on rookery location and accessibility to foraging grounds. There is a lack of data to allow a SWIO analysis of reproductive parameters that could validate our theoretical results regarding reproductive output. However, these variations are in accordance with sea turtle reproductive biology. For example, Troeng and Chaloupka (2007) suggested that short remigration intervals (2–3 years) observed in the rookery of Costa Rica could be due to the relative proximity of the foraging sites.

We also demonstrated that overall reproductive output heavily relied on spatial and environmental conditions, and that, under these conditions, behavioral strategies might perform differently essentially by affecting the energetic level at nesting (Figure S1). Oceanic currents introduced environmental heterogeneity and uncertainty along migration pathways generally lowering the overall reproductive output. Under such conditions, the model predicted that a “stayer” foraging behavior led to better reproductive output at population level. This suggested that uncertainties along the way during migration might favor fidelity to foraging grounds, a commonly observed behavior for sea turtles (Broderick et al., 2007; Godley et al., 2008). Although at a global scale, the flow of currents is predictable, this is not so from the individual's point of view that cannot be sure which currents it will face during migration. In our model, the currents introduce energetic constraints or savings, but the impact on the turtle's energetic level, upscaled to the population level, will emerge from the model. Costs of searching for forage might be a risky behavior leading to overall lower reproductive output, especially when neighboring foraging patches are of low quality or have been exploited already by other turtles.

Likewise, the model predicted that more “investment” in reproduction was preferable when perturbation took place. Testing this hypothesis in the field is challenging. However, for the leatherback population of French Guyana, it has been demonstrated that a trade‐off exists between the reproduction effort and the delay between reproduction events (Rivalan et al., 2005), suggesting that larger reproductive intervals could be counterbalanced by higher investment in reproduction.

Interestingly, while favorable behaviors were identified under various environmental circumstances, no general “best strategy” arose from the model results. Under identical parameters, responses of reproductive output to behavioral strategies could be opposite depending on the location of considered rookeries and therefore influence by other extrinsic factors. Landscape configuration can lead to nontrivial responses, which underlines the importance of explicitly considering space and movement.

### 4.2 Migration corridors, foraging hot spots, and conservation of green turtle in the SWIO

Under multiple scenarios, the model highlighted two connected provinces, in the north and the south of the Mozambique Channel, with their own structural particularities in regard to green turtle populations. The model provided some explanations to the origin of this regional pattern and also allowed drawing further hypotheses. The conclusions and their comparison with field observations are discussed in the following, as well as potential conservation implications.

#### 4.2.1 Migration and foraging hot spots, relative contribution of regional rookeries

A northern migration corridor emerged in the model between the north of Madagascar and the northern coast of Mozambique. From the model, we could infer that the presence of numerous nesting sites in this area and their central location relative to the distribution of the regional feeding patches (Figure 2) is likely to explain the high densities of migrating turtles in this area. Considering oceanic currents (scenario 2), we found that the westward North‐Eastern Madagascar Current (NEMC) tended to widen the northern migration corridor along its east–west axis. This might explain that a majority of tracked nesting green turtles from Tromelin migrated along the NEMC current (Dalleau, 2013). In the Southern part of the Mozambique Channel, a trident shaped migration corridor was also observed, with a high level of frequentation due to the high number of females nesting in Europa Island (Bourjea, 2015). The existence of similar migration corridors was also one of the major observations of a regional tracking study (Dalleau, 2013).

According to the model, the coastal areas of Africa and Madagascar bordering the north of the Mozambique Channel would be the most frequented one by turtles originating from numerous rookeries. This is consistent with known foraging locations from field observations (Fulanda et al., 2007; Muir, 2005; Okemwa et al., 2004). Further, this is also in agreement with the distribution of foraging areas of turtles' satellite‐tracked from the majority of the regional nesting sites (Dalleau, 2013) that identified four regional foraging hot spots of which three are bordering the north of the Mozambique Channel in Tanzania, northern Mozambique, and northern Madagascar. It is worthwhile to mention that the choice of feeding patches was not imposed throughout a simulation but emerged from turtle's decisions depending on density and habitat quality. This indicates that in our model the mechanisms of habitat selection are working well.

Another conclusion of the model was that numerous rookeries contribute to the nesters composition of the northern part of the region (5–15°S), while the high level of frequentation of the southern part (18–25°S) relies on a single rookery, Europa. This result is in agreement with regional genetic analysis based on mitochondrial DNA. Indeed, Taquet (2007) showed that foraging adult green turtles of Tanzania and western Madagascar share haplotypes mostly observed in the northern nesting sites (Bourjea, 2015; Bourjea, Lapègue, et al., 2007b), while foraging adults of South Africa and stranded adults of the southwest of Madagascar share haplotypes mostly observed in Europa nesting population (Bourjea, Lapègue, et al., 2007b).

#### 4.2.2 Implications for conservation

The model promoted areas as major regional migratory and foraging hot spots for adult female green turtles. Two provinces, in the north and south of the Mozambique Channel, with contrasted dynamics were characterized. The particularities of each lead to different challenges in terms of conservation.

In the SWIO, direct take and coastal fisheries bycatch are a major threat (Bourjea, 2015). Looking at the model results, we could infer that high levels of bycatch and direct take reported along the east African coasts (see review in Bourjea, 2015) of Mozambique (Gove, Pacules, & Gonçalves, 2001; Kiszka, 2012; Williams, 2017; Williams, Pierce, Hamann, & Fuentes, 2019), Tanzania (Moore et al., 2010; Muir, 2005), and Kenya (Mueni & Mwangi, 2001; Okemwa et al., 2004) might probably affect all sea turtle nesting populations of the region, and more specifically nesting populations from the north of the Mozambique Channel.

High level of direct take also occurs in the western coast of Madagascar (Rakotonirina, 2011). A majority of individuals are green turtles captured along the southwest coast (Humber et al., 2011). The model showed, in agreement with genetics data (Taquet, 2007), that adult individuals of western Madagascar were essentially issued from the nesting population of Europa Island. Conservation efforts in this area would then consequently benefit preferentially Europa Island's green turtle nesting population.

The model, under a perturbation scenario, also pointed out that depleting foraging grounds of the southern Mozambique Channel might raise the pressure on northern foraging grounds and indirectly have consequences on the reproductive output of rookeries at regional scale. As a consequence, while protection efforts might target specific areas depending on the conservation goals, the model clearly highlighted that green turtle conservation should be approached as a regional matter.

As proposed by Wallace, DiMatteo, et al., 2010a, our model results confirm that the “Regional Management Unit” identified for green turtles in the SWIO (Wallace, DiMatteo, et al., 2010a) is the best scale for this area, but the model substantially highlighted the existence of subregions or provinces with distinct but connected population dynamics. If the Regional Management Unit can be apprehended as a whole the variable contribution from nesting to regional migration and feeding hot spots proves that localized conservation actions will not affect populations in the same way. Understanding more precisely the regional spatial dynamics of green turtle is precious to conduct monitoring and conservation efforts where they are most needed within the Regional Management Unit.

### 4.3 Outlook on model improvements

Building the model provided valuable insight into the areas for which biological information is available, but also into the areas for which there are critical gaps in species biological and ecological knowledge. Sea turtles' spatial ecology benefits the recent progress in biotelemetry and more particularly in satellite tracking. There is now a better understanding on the spatial ecology of sea turtles in the major oceans (Hays, 2008) and its long distances migrations related to oceanic environment (Luschi, 2013). In the western Indian Ocean, recent results using genetics (Bourjea, Lapègue, et al., 2007b; Bourjea, Mortimer, et al., 2015b), satellite tracking (Dalleau, 2013), and spatial statistics (Dalleau et al., 2012) have provided a better understanding of the regional dynamics of green turtle in the region. High‐density tracking data can also be used to develop correlative habitat models (often also referred to as species distribution models), which predict high‐quality habitat; this has been done already for the loggerhead sea turtle (Abecassis et al., 2013).

The concept of integrating movement, energetics, and reproduction is novel for this system, and confirmed important areas for conservation. We propose linking the physiology of the animal and its physical environment (food and currents in our case) as a way forward in understanding movement decisions and emerging population patterns; this also offers new predictive tools to assess effects of habitat or climate change (Malishev, Bull, & Kearney, 2018). Historically, metabolic physiology studies have used respirometry to assess metabolic rates in closed‐circuits systems and doubly labeled water technique has also been used to estimate field metabolic rates (Enstipp et al., 2016; Wallace & Jones, 2008). The latest advances in techniques such as accelerometry might also provide better insights (Hays et al., 2016). For various sea turtle species, there has been increasing knowledge about energetic balance of specific physiological states: nesting (e.g., Hays, Broderick, Glen, & Godley, 2002a), migration (Enstipp et al., 2011; Halsey, Jones, Jones, Liebsch, & Booth, 2011), or foraging (e.g., Ballorain et al., 2013; Ballorain et al., 2010; Enstipp et al., 2016). Still it remains a challenge for measuring metabolic rates of free‐ranging turtles, and no integrative eco‐physiological model exists yet that encompasses and unifies the three processes together (Williard, 2013). Additionally, the physiological factors that trigger nesting migration at the individual level are still poorly understood. Progress in energetics of sea turtles would be of key value to improve individual‐based modeling of sea turtles' ecological processes in their environment from basic principles. An additional way of improving the sea turtles' energy budgets in the model would be using Dynamic Energy Budget (DEB) theory (Kooijman, 2010), which is a generic model that predicts how much an animal invests energy in growth, maintenance, and reproduction, and how this depends on the animals size and maturity. DEB is increasingly used in individual‐based models (Galic, Grimm, & Forbes, 2017; Galic, Sullivan, Grimm, & Forbes, 2018; Martin et al., 2013; Martin, Zimmer, Grimm, & Jager, 2012) and is also under development for improving the energy budget model of a model of harbor porpoise; C. Ghallager, personal communication. While the original DEB theory does not explicitly address movement, this has been added recently for the movement of lizards (Malishev et al., 2018).

In the SWIO, population trends have been in most cases estimated from nesting crawls (Bourjea, Dalleau, et al., 2015a; Bourjea, Frappier, et al., 2007a; Lauret‐Stepler et al., 2007; Mortimer, 2012; Mortimer, Brandis, et al., 2011b; Mortimer, Camille, & Boniface, 2011a) and individual's reproductive parameters have rarely been monitored, as nesting sites are hardly accessible in this region. Spatial comparison of individual reproductive parameters would be required for a better assessment of population's viability as we showed that response to environmental uncertainties such as oceanic current or perturbations varied according to nesting and foraging strategies. Future implementation of the model should therefore also include demographic processes. Indeed, while reproductive potential was considered, survival and fecundity were in fact not explicitly implemented in our model. Foraging and nesting strategies were fixed for a given simulation. This is unlikely to be the case in reality since various strategies probably evolve or coexist. Ideally, decision strategies should emerge from the model. This would require adaptation and survival to be also implemented.

Implementing perturbations enabled us to qualitatively show the potential of IBMs to predict spatial, temporal, and survival consequences of modifications of the foraging environment. Advanced modeling could provide an effective tool to predict the impact of climate change on sea turtles' populations as spatial complexity of their life cycle makes prediction hardly accessible (Hawkes, Broderick, Godfrey, & Godley, 2009). Regarding more direct human perturbation, there is still little literature about poaching and artisanal fisheries bycatch in the region (Bourjea, 2015; Humber et al., 2011; Temple et al., 2018). Including human threats quantitatively in the model would make it a perfect tool for managers and decision makers.

### 4.4 Behavioral and spatially explicit modeling for sea turtles: the quest for the grail

Our study underlined the importance of spatially explicit modeling to spatial ecology and population dynamics of migratory species. The model provides new insights on green turtle biology, linking spatial ecology, and population dynamics through the use of a basic physiological energy budget and behavioral decision strategy model. We integrated in a spatially realistic context the three main processes of the adult biology of sea turtles: reproduction, migration, and foraging. While it remained at this stage a conceptual and explorative approach, the main benefits were to (a) provide an operational tool to characterize the spatial structure of green turtle populations at regional scale and to (b) explicitly explore the role of landscape configuration (nesting and foraging site distribution, terrestrial barriers such as Madagascar, environmental drivers such as oceanic currents) and (c) individual's decision strategies on sea turtle spatial ecology. Practical conclusions provide important consideration that addresses large research priorities recently identified for sea turtles (Rees et al., 2016).

With the improving knowledge on sea turtle biology at individual scale, individual‐based approaches should progress and become more integrative. Such knowledge may allow highlighting different individual foraging and/or nesting strategies that may be tested in the model by implementing adaptiveness of fixed versus plastic responses to environmental changes (e.g., Bradshaw, Hindell, Sumner, & Michael, 2004; Railsback & Grimm, 2019). The next logical step to improve these models requires a better ability to explicitly consider landscape and movement in a realistic context. The model presented here constitutes a first step. Although explorative, some of the ideas implemented should inspire spatial ecologist aiming at unifying movement ecology and population dynamics.

## CONFLICT OF INTEREST

None declared.

## AUTHOR CONTRIBUTIONS

MD developed, programmed, and analyzed the model and led the writing of the paper. SK‐S and VG codeveloped the model and contributed to writing the paper. JB, YG, and GL provided input in terms of expert knowledge about the species and system addressed and contributed to writing the paper.

## Data Availability

All data required to run the model, that is, input files with the location of foraging and nesting sites as well as all input maps, are available, together with the NetLogo program implementing the model, the ODD model description, and instructions for running the model, in the Computational Model Library of the ComSES Network: https://www.comses.net/codebases/69863caa-2f8e-4412-a564-a2826d9d38d3/releases/1.0.0/.
