# Supplementary material for: Modeling the emergence of migratory corridors and foraging hot spots of the green sea turtle
Source: Ecol Evol. 2019 Aug 18;9(18):10317–42. doi: 10.1002/ece3.5552 (PMC6787826; doi:10.1002/ece3.5552)
Supplement: Supplementary file 1 [file ECE3-9-10317-s001.docx]

# Supplement

# Energy at nesting

In the absence of oceanic currents (scen. 1), energy level at nesting was not influenced by either nesting allocation or foraging patch fidelity strategy to an extent that would be biologically meaningful (Fig. B.1a).

When considering oceanic currents (scen. 2; Fig. B.1b) independently from nesting and foraging strategies, the energy level at nesting was drastically lower than in the absence of currents (scen. 1). Oceanic currents introduce environmental uncertainties and increased migration duration, leading to lower energy levels at nesting. Nevertheless, the decrease in energy levels was strongly limited for ‘stayer’ foraging tendencies and more important for ‘mover’ foraging tendencies. In addition, we observe higher levels of variations in the energy levels for ‘mover’ tendencies. Regarding nesting strategies, the decrease was also slightly smaller for ‘conservative’ nesting strategies than ‘investment’ nesting tendencies.

In the third scenario (scen. 3), introducing human perturbations in the southern feeding patches did not affect the overall energy levels at nesting (Fig. B.1c). ‘Invester’ nesting tendencies nevertheless lead to higher variability in nesting energy levels in comparison to scenario 1.


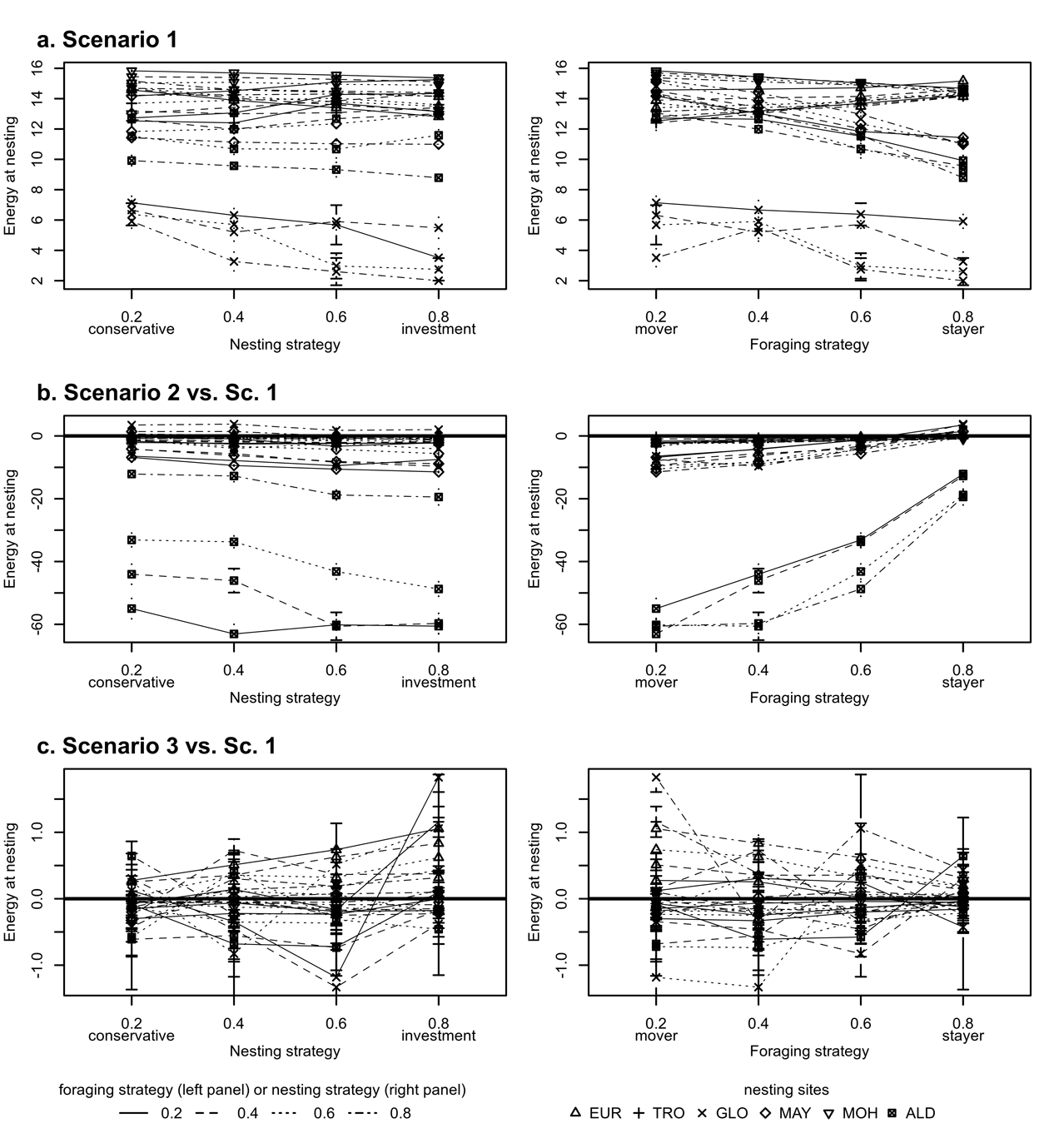


Fig. S1. Mean individual energy level after nesting: (a) energy levels for scenario 1, (b) energy levels for scenario 2 relative to scenario 1 and (c) energy levels for scenario 3 relative to scenario 1. Error bars respectively correspond to standard errors of energy levels respectively for (a) scenario 1, (b) scenario 2 and (c) scenario 3. Line type represents foraging strategy (left panel) or nesting strategy (right panel) taken in (0.2; 0.4; 0.6; 0.8). Point types represents the 6 main nesting sites (EUR: Europa, TRO: Tromelin, GLO: Glorieuses, MAY: Mayotte, MOH: Mohéli, ALD: Aldabra).

# Remigration interval

Mean individual remigration intervals (duration between two successive nesting cycles) over the entire set of simulations ranged from 1.9 to 7.1 years. While there is no estimation of remigration intervals in the population of the South West Indian Ocean, these values fall within the range observed worldwide which varies between 2 to 7 years (see review in Troeng and Chaloupka 2007).

Not surprisingly, remigration interval was directly impacted by nesting strategy (Fig. B.2a, left panel). Under scenario 1 (Fig. B.2a, left panel), higher investment tendencies (0.8) lead to remigration intervals comprised between 6.48 and 7.20 years while remigration intervals for more conservative tendencies (0.2) was only comprised between 2.31 and 2.55 years. More energy being required for ‘investment’ tendencies, foraging lasted longer for these strategies. Foraging strategy, on the other hand, had little impact on mean remigration interval (Fig. B.2a, right panel).

When considering ocean currents (scen. 2), remigration interval dropped down slightly to 4.13 ± 0.16 years. Perturbations (scen. 3) did not have a strong impact on the mean remigration interval (4.41 ± 0.16 years). Trends in remigration intervals under scenario 2 and scenario 3 along nesting or foraging strategy did not differ much from scenario 1 (Fig. B.2b and c). In summary, while oceanic currents lower and perturbations raise the mean remigration interval, decision strategies did not have major consequences on these variations.


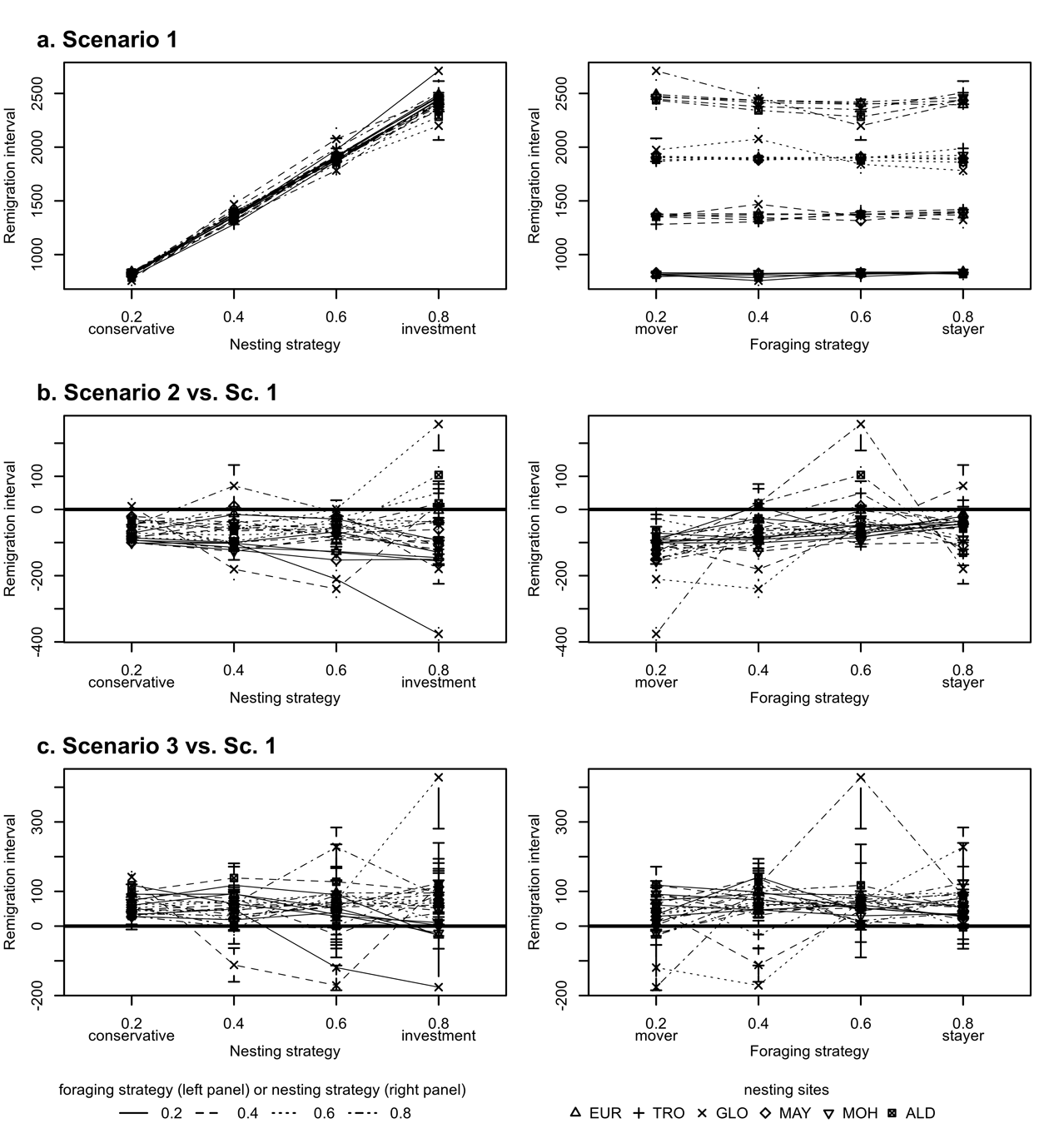


Fig. S2. Mean individual remigration interval (days), time difference between two nesting phases. (a) Remigration intervals for scenario 1, (b) remigration intervals for scenario 2 relative to scenario 1 and (c) remigration intervals for scenario 3 relative to scenario 1. Error bars respectively correspond to standard errors of remigration intervals respectively for (a) scenario 1, (b) scenario 2 and (c) scenario 3. Line type represents foraging strategy (left panel) or nesting strategy (right panel) taken in (0.2; 0.4; 0.6; 0.8). Point types represents the 6 main nesting sites (EUR: Europa, TRO: Tromelin, GLO: Glorieuses, MAY: Mayotte, MOH: Mohéli, ALD: Aldabra).

# Reproductive output

**
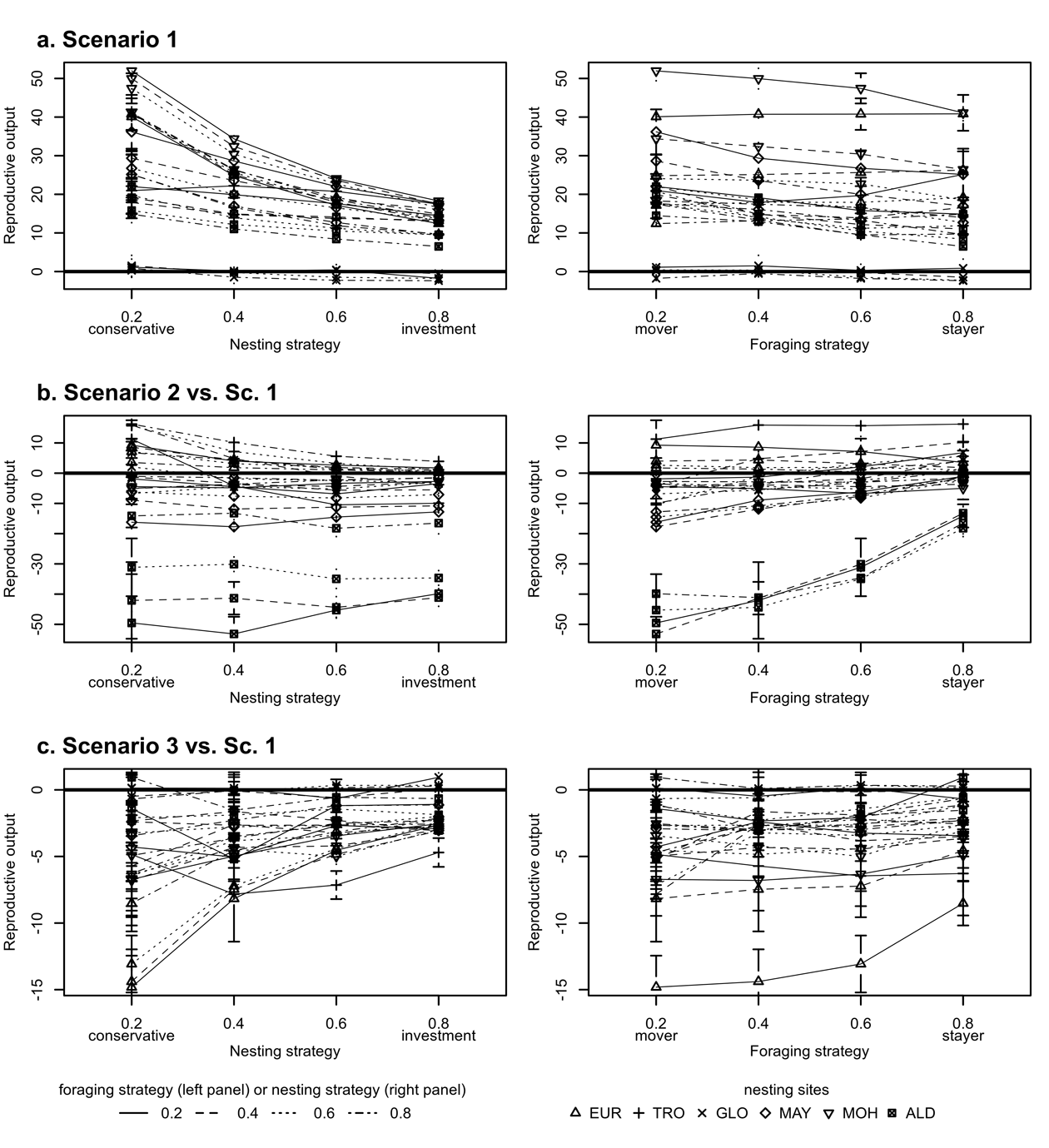
**

Fig. S3. Overall reproductive output at nesting sites depending on nesting and foraging strategies. Overall reproductive output for a given nesting site is directly proportional to individual’s energy level after nesting and inversely proportional to individual’s remigration interval. (a) Reproductive outputs for scenario 1, (b) Reproductive outputs for scenario 2 relative to Scenario 1 and (c) Reproductive outputs for scenario 3 relative to scenario 1. Error bars respectively correspond to standard errors of reproductive outputs respectively for (a) scenario 1, (b) scenario 2 and (c) scenario 3. Line type represents foraging strategy (left panel) or nesting strategy (right panel) taken in 0.2, 0.4, 0.6 or 0.8. Point types represents the 6 main nesting sites (EUR: Europa, TRO: Tromelin, GLO: Glorieuses, MAY: Mayotte, MOH: Mohéli, ALD: Aldabra).
